# Cu–Ga Interactions and Support Effects in CO_2_ Hydrogenation to Methanol Catalyzed by Size-Controlled CuGa Nanoparticles Deposited on SiO_2_ and ZnO

**DOI:** 10.1021/acscatal.5c03414

**Published:** 2025-10-02

**Authors:** David Kordus, Janis Timoshenko, Núria J. Divins, See Wee Chee, Eduardo Ortega, Mauricio Lopez Luna, Uta Hejral, Ane Etxebarria, Beatriz Roldan Cuenya

**Affiliations:** † Department of Interface Science, 28259 Fritz-Haber Institute of the Max Planck Society, Berlin 14195, Germany; ‡ Department of Physics, Ruhr University Bochum, Bochum 44801, Germany

**Keywords:** CO_2_ hydrogenation, methanol, NAP-XPS, EXAFS, Cu, Ga

## Abstract

Growing environmental concerns have led to a need for the reduction of CO_2_ emissions and the search for alternative fuels. The synthesis of methanol via the CO_2_ hydrogenation reaction provides a promising approach for these tasks. Promoting the existing Cu-based catalysts with Ga might be an option to create more effective catalysts. Here, size-controlled bimetallic CuGa nanoparticles (NPs) supported on either SiO_2_ or ZnO were synthesized to study the nature of the interaction of Cu and Ga. Operando spectroscopy and diffraction characterization methods (XPS, XAS, XRD) were employed to establish structure, chemical composition, and reactivity correlations. We find that Ga stays oxidized under the reaction conditions and segregates to the surface. For the CuGa NPs/ZnO, the dominating interaction of Cu with ZnO inhibits the promoting effect of Ga. Only on the inert SiO_2_ support, the beneficial influence of Ga is visible. Furthermore, high pretreatment temperatures were found to result in a favorable Cu–Ga interaction by partially reducing Ga, which is beneficial for methanol selectivity.

## Graphical abstract

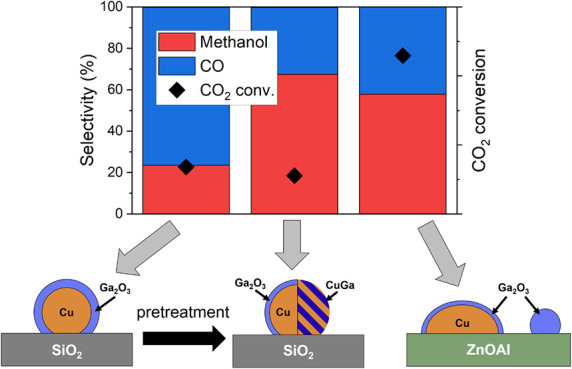

## Introduction

Methanol is one of the most important chemicals in the world that is used both as an ingredient in many products and as a building block for other chemicals. Methanol is also an important energy carrier candidate in alternative energy schemes that aim to produce sustainable fuels using recycled CO_2_ and green hydrogen.^1^ Currently, the commonly employed industrial catalyst for methanol synthesis is a ternary catalyst consisting of Cu, ZnO, and Al_2_O_3_ with Cu as the active material,^2^ but the high pressures (50–100 bar) needed for the selective production of methanol limit the efficiency of the conversion process. Especially in a gas feed consisting only of CO_2_ and H_2_, the traditional Cu/ZnO/Al_2_O_3_ catalyst displays lower methanol selectivity because of the competing reverse water gas shift (RWGS), which produces CO and water.^3^ In particular, the presence of water can limit the effectiveness of the catalyst.^4^

A strategy to increase the methanol yield and selectivity is to modify the catalyst with an additional component. A good candidate for this would be gallium. Several approaches were taken in the past to incorporate Ga into a methanol synthesis catalyst. For instance, Ga_2_O_3_ has been suggested as a substitute to the traditional ZnO for Cu particles.^5,6^ Alternatively, Ga has also been alloyed with other metals, forming bimetallic catalysts such Pd_2_Ga,^7–9^ Ni_5_Ga_3_,^10–13^ or PtGa,^14^ which were found to be excellent material systems for CO_2_ hydrogenation to methanol at lower pressures than the Cu/ZnO/Al_2_O_3_ system. Furthermore, adding Ga to the existing Cu/ZnO system was also found to increase the methanol yield^15–17^ and selectivity.^17–19^ Similar effects were also observed when adding Ga to Cu/ZrO_2_ ^20,21^ and Cu/ZnO/ZrO_2_ ^22,23^ catalysts. However, the reasons behind the Ga-driven improvement of the selectivity during CO_2_ hydrogenation toward methanol remain under discussion. The difficulty here lies in untangling the complex morphological, structural, and compositional changes that such a multicomponent catalyst undergoes in the course of the catalytic reaction. Several papers claim an improvement of the conversion, methanol yield, or selectivity upon addition of Ga to the catalyst. The reasons given for these improvements were a higher catalyst surface area,^24,25^ the prevention of Cu aggregation,^17,21,26–28^ an enhanced reducibility of the catalyst,^17,18^ a better CO_2_ ^29,30^ or H_2_ ^31,32^ adsorption capacity, and activation. Furthermore, it was proposed that Ga leads to the formation of additional sites^19,33^ that are highly selective for methanol synthesis rather than for the RWGS reaction or that the insertion of Ga can modify the ZnO support by changing its reducibility and defect site density,^16^ which leads to a higher methanol yield and selectivity. However, the specific influence of Ga promotion on the catalyst strongly depends on the chemical state and structure in which Ga is present. For that matter, Ga can be reduced and form an alloy,^34^ be present as Ga^3+^ species,^16,34,35^ or form a spinel^18,21,35^ (e.g., CuGa_2_O_4_ or ZnGa_2_O_4_). At the same time, the Cu-to-Ga ratio^19,34^ can influence the state of Ga (similar to ZnO in the traditional Cu/ZnO system^36,37^ and thus also modify its catalytic performance.

In this work, we have synthesized size-controlled bimetallic CuGa nanoparticles (NPs) using colloidal methods (inverse micelle encapsulation) and dispersed them on either SiO_2_ or ZnO supports. Thus, we reduced the complexity of the system to better focus on the interaction between Cu and Ga. SiO_2_ as an inert support is not expected to participate in the reaction, and thus, we were able to directly study the Cu–Ga interaction. ZnO is used as a reference support to gain insight into the promotional effect of Ga on the Cu/ZnO system, which is most frequently used for methanol synthesis. The materials’ gap has been closed in this study by depositing nearly identical (size, shape, and composition) CuGa NPs on two different high-surface-area nanocrystalline powder supports as well as on single crystals [SiO_2_/Si­(111) and ZnO(0001)], which are more amenable for characterization with traditional surface science methods. Additionally, the pressure gap has been partially bridged by combining near-ambient-pressure X-ray photoelectron spectroscopy (NAP-XPS), an excellent tool to study surface segregation phenomena,^38–40^ with *in situ* X-ray absorption spectroscopy (XAS) experiments at industrially relevant high pressures. The spectroscopic characterization here thus covers a range from the mbar range (XPS, ∼1 mbar) to atmospheric pressure (XAS, 1 bar). The synergistic multitechnique characterization approach employed here sheds light into the role of Ga on the improved CO_2_ hydrogenation performance of CuGa systems.

## Experimental Section

### Catalyst Preparation

CuGa particles were synthesized by an inverse micellar encapsulation method following a procedure described previously.^38,39^ The target Cu-to-Ga ratio was 70:30, although, as shown below, the actual Ga content in the prepared catalyst is significantly lower. For the NPs deposited on flat supports, the metal precursors used for the synthesis were CuCl_2_·2H_2_O (Sigma-Aldrich, ≥99.95%) and Ga_2_Cl_4_ (Sigma-Aldrich, 99.999%) salts. The diblock copolymer used was poly­(styrene)-*block*-poly­(2-vinylpyridine) (PS-P2VP) (Polymer Source Inc.), with a PS:P2VP ratio of 26000:4800. The micellar NPs were deposited on the planar SiO_2_/Si­(111) and ZnO(0001) substrates by dip-coating (speed 1 cm/min). Hereby, the Si(111) crystal has a native oxide layer (SiO_2_), whose thickness was additionally increased by the subsequent O_2_ plasma treatment employed to remove the polymer from the micellar NPs. The polymer was etched by an O_2_ plasma treatment (20 min, 20 W, 0.45 mbar, SPI Plasma Prep III Plasma Etcher).

For the CuGa NPs deposited on nanocrystalline oxide powder supports, the micelle synthesis used Cu­(NO_3_)_2_·3H_2_O (Sigma-Aldrich, 99–104%) and Ga­(NO_3_)_2_·*x*H_2_O (molecular weight: 255.74 g/mol, Sigma-Aldrich, 99.999%) salts. The PS:P2VP ratio of the polymer used was 130000:135000 (Polymersource Inc.). The resulting micellar NPs were deposited on SiO_2_ (STREM Chemicals) and ZnO/Al_2_O_3_ (synthesis procedure reported elsewhere)^40^ powders by incipient wetness impregnation, with a target ratio of 5% of (Cu + Ga)/support by mass. In these samples, the polymer was removed by calcination in a 20% O_2_ in an Ar mixture at 420 or 440 °C for 6 h on the SiO_2_ or ZnOAl support, respectively. The optimal calcination temperature for polymer removal was determined by thermogravimetric analysis (TGA). For simplicity, the NPs deposited on the ZnO/Al_2_O_3_ support will be referred to as ZnOAl-supported in the rest of this work since Al_2_O_3_ makes only a small fraction (10 mol %)^40^ of the support material. The ZnOAl support was chosen for multiple reasons. First, the same support was previously used for our study on pure micellar-synthesized Cu and CuZn NPs^40^ and therefore allows us to correlate our results with those from the previous study. Furthermore, the commercial catalyst also consists of Cu, ZnO, and Al. Thus, the CuGa NPs supported on ZnOAl resemble the traditional catalyst with the additional Ga component. In the industrial catalyst, Al_2_O_3_ serves as a structural promoter, helping to prevent sintering of the catalyst or Cu and Zn alloying.^40^ On the other hand, as SiO_2_ is (mostly) inert in this reaction, the CuGa NPs supported on SiO_2_ allow us to study solely the CuGa interaction without the possible promoting influence of the support.

### Catalyst Characterization

**AFM:** characterization of the size of the NPs supported on the flat substrates was done with atomic force microscopy (AFM). AFM was performed on the plasma-cleaned samples with a Bruker Multimode 8 microscope in the tapping mode. Data analysis of the NP height was done with the open source software Gwyddion.^41^

**STEM:** characterization of the size and distribution of the NPs supported on the powder catalysts was done via scanning transmission electron microscopy (STEM) with a FEI Titan 80–300 microscope. The samples were measured before and after the reaction. For characterization after the reaction, the powder was transferred from the reactor to a N_2_-filled glovebox where the sample was prepared and loaded into a vacuum transfer holder (Gatan). This way, the sample could be measured in the TEM microscope without exposure to air, to avoid modifications of the catalyst’s structure.

**ICP:** the composition of the NP samples deposited on the oxide powders was determined by inductively coupled plasma-mass spectrometry (ICP-MS, iCAP RQ ICP-MS from Thermo Scientific). Prior to the measurements, the samples were dissolved in a mixture of 2 mL H_2_SO_4_, 2 mL HNO_3_, and 6 mL HCl. This solution was then digested in a microwave (Anton Paar GmbH, Multiwave GO) for 30 min at 180 °C and afterward further diluted with water for the final ICP measurement.

**TGA:** thermogravimetric analysis (TGA) was employed for the determination of the optimal calcination temperature required for the complete polymer removal from the powder samples. Thus, the sample weight was measured, while the sample was heated from room temperature to 600 °C in a 20% O_2_ in a N_2_ mixture (synthetic air). The weight loss during this procedure is linked to the decomposition of the polymeric ligands, and the calcination temperature was chosen slightly higher than the highest weight loss peak.

### Catalytic Performance

The performance of the catalysts was tested in a packed-bed flow reactor. For each measurement, around 50 mg of the catalyst was used and diluted with SiC in a 1:6 ratio. The catalyst was fixed in position in a glass-lined steel reactor tube with two quartz wool plugs. Reduction of the catalyst was done in a 10% H_2_ + 90% He mixture with a total flow of 50 mL/min at either 250 or 500 °C. Afterward, the catalytic performance was evaluated in a 60% H_2_ + 20% CO_2_ + 20% He mixture. Hereby, He served as an internal standard for the measurement. The total flow in all experiments was 25 mL/min, except for the comparison of Cu, CuGa, and CuZn NPs deposited on SiO_2_. Those experiments were performed with a total flow rate of 17 mL/min. Data were collected at atmospheric pressure and at 20, 40, and 60 bar. The temperature during the reactivity measurements was 250 °C. The catalysts were kept at each reaction condition for at least 12 h to ensure that they have reached a steady-state operation without changes in their activity. The product analysis was done by online gas chromatography (GC) with an Agilent Technologies 7890B gas chromatograph equipped with a flame ionization detector (FID) and two thermal conductivity detectors (TCDs).

A note of caution here is that CO, produced by the RWGS, cannot be properly quantified for the pure Cu NPs under the applied conditions because of the low conversion rates and the fact that in GC, CO is detected with a TCD, which is less sensitive than the FID detector used for the quantification of CH_4_ and methanol. For this reason, CO being produced by the RWGS is excluded from the selectivity plots for that experiment, and only the products coming from the CO_2_ hydrogenation reactions (CH_4_ and CH_3_OH) are shown.

**XAS:** X-ray absorption spectroscopy (XAS) experiments were performed at the CLAESS beamline at the ALBA synchrotron. For *operando* measurements, a multipurpose solid–gas reactor^42^ was used. The powder samples were diluted with BN (boron nitride) and then pressed into pellets for optimal signal. The Cu K-edge (8979 eV) and Ga K-edge (10367 eV) XAS data were collected in the fluorescence mode using a 6-channel Si drift detector. For energy selection, we used a fully tuned Si(311) monochromator, and a reflective mirror was used to reject the higher harmonics. The X-ray intensity of the incident beam was measured using an ionization chamber with a 97.4% N_2_ and 2.6% Kr mixture. Sample loading was optimized to ensure maximum fluorescence intensity at the Ga K-edge. As a result and due to the low loading of Ga compared to Cu, the Cu K-edge XAS spectra showed significant distortions due to the self-absorption effect. Therefore, only the spectra of the Ga K-edge were used for quantitative analysis.

Reference XAS measurements for CuO and Ga_2_O_3_ phases were performed in the transmission mode for the commercial CuO and Ga_2_O_3_ powder samples pressed into a pellet. The reference spectrum for metallic Ga was taken from our previous work.^12^ XAS data extraction and alignment were performed with the Athena software package.^43^ For EXAFS data fitting, we relied on nonlinear least-squares fitting as implemented in the FEFFIT code. Linear combination analysis (LCA) of *in situ* Ga K-edge XANES spectra was performed using in-house-developed Wolfram Mathematica scripts. Spectra collected for our catalyst in air and for metallic Ga were used as references.

**XPS:** NAP-XPS measurements were performed at the HIPPIE beamline at the MAX IV laboratory. The measurements were done with two different photon energies to extract depth-dependent information due to the different inelastic mean free path (IMFP) of the electrons. The IMFP values for the used photon energies of 1320 and 1540 eV were calculated with the QUASES software, which uses the Tanuma, Powell, and Penn algorithm (TPP2M).^44^ The resulting IMFP values are 0.78 and 1.23 nm for Ga and 0.79 and 1.07 nm for Cu at 1320 and 1540 eV, respectively. Quantitative fitting of the 2p regions was done following previously described peak shapes and positions.^45,46^ The energy scale of all spectra was aligned to the support peak positions of ZnO or SiO_2_ under the corresponding conditions.

In order to change the atmosphere in the sample chamber, the sample was cooled down to room temperature; then, the gases were pumped out, and a new gas mixture was introduced before heating up the sample again.

**XRD:** X-ray diffraction (XRD) patterns of the catalysts were recorded with a Bruker-AXS D8 Advance diffractometer equipped with a Cu Kα source and a position-sensitive energy-dispersive LynxEye XE-T detector. The diffraction patterns were recorded in a 2θ range from 10 to 90° in 0.02° steps. Data analysis was performed with the DIFFRAC.EVA software package (Bruker).

## Results and Discussion

### Morphological and Structural Characterization

The coverage and size distribution of the CuGa NPs on the planar samples after polymer plasma removal were studied by AFM (Figure 1A and D). Analysis of the AFM images returned an average particle size, here given as NP height, of 4.3 ± 1.3 nm and 2.9 ± 0.8 nm for the NPs supported on SiO_2_/Si­(111) and ZnO(0001), respectively. Figure 1C and F show the corresponding height histograms. The NPs have different average size distributions depending on the support materials, despite the fact that they were prepared from the same NP colloidal solution. This indicates a different interaction of the particles with the support, which is stronger for CuGa/ZnO, and thus, flatter NPs (lower height than on SiO_2_) were observed. The mean particle size was measured again after the reaction (Figure 1B and E), and a reduced size of 2.5 ± 1.2 nm and 2.4 ± 0.9 nm was found for the SiO_2_ and ZnO-supported samples, respectively.

**Figure 1:**
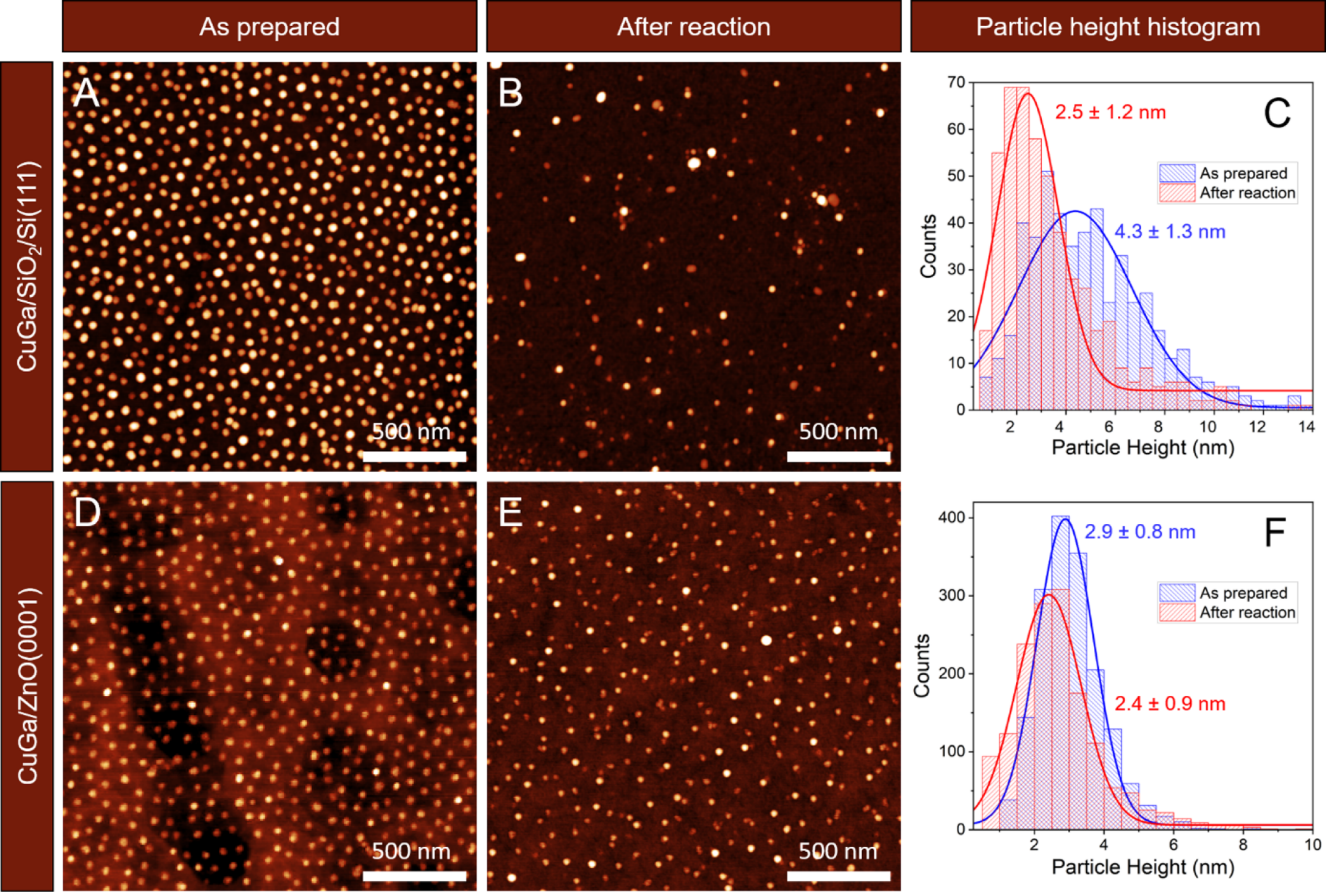
AFM images of the Cu_70_Ga_30_ NPs supported on (A–C) SiO_2_/Si­(111) and (D–F) ZnO(0001), after O_2_-plasma for polymer removal (A, D), after CO_2_ hydrogenation during NAP-XPS measurements (B, E), and the corresponding NP height histograms (C, F). The histograms were compiled from the analysis of multiple AFM images.

For the catalytic studies, identical size-controlled CuGa NPs were also impregnated on SiO_2_ and ZnOAl nanocrystalline powder supports by incipient wetness impregnation. ICP showed a Cu/Ga ratio of about 9/1 in the as-prepared catalyst (Table S1). The total ratio of Cu and Ga compared to the support is about 3%. The low loading is needed to get well-defined small particles dispersed on the powder supports. XRD showed only peaks originating from the ZnOAl support, CuO (Tenorite), or the diffuse scattering of SiO_2_. The corresponding XRD patterns are shown in Figure S1. The dominating support features on ZnO are expected due to the small size of the CuGa particles employed and their low loading (3%) on the support. Therefore, only small peaks corresponding to the main Cu component of the NPs are visible. As expected, the NPs appear fully oxidized (CuO) due to the calcination treatment in O_2_ to remove the polymer from the NP synthesis. For the SiO_2_-supported sample, multiple CuO (e.g., 34.8°, 38.2°, and 48.2°) peaks can be clearly identified. In contrast, for the particles supported on ZnOAl, only the main peak of the CuO structure at 38° is clearly seen. Almost all other CuO reflections are at similar positions as the peaks from ZnO and are therefore buried under the much stronger contribution from the ZnO support. Peaks corresponding to Ga_2_O_3_ cannot be observed. The lack of a Ga signal detected by XRD in the as-prepared Cu_90_Ga_10_ NPs is assigned to their small size and low Ga content as well as the likely amorphous nature of GaO_*x*_ present at this preparation stage. The absence of a Ga contribution in the XRD pattern because of its high dispersion and disorder is a frequently reported phenomenon.^17,19,20,35^ However, the peak appearing at 44.4° for the CuGa/SiO_2_ sample has been previously assigned to either CuGa_2_ ^47,48^ or CuGa_2_O_4_ spinel structures.^49,50^ Nevertheless, the possibility of forming a CuGa_2_ alloy during the preparation is highly unlikely because the calcination process that is part of the catalyst preparation is done under an atmosphere containing oxygen. Thus, the formation of CuO and Ga_2_O_3_ or a CuGa_2_O_4_ spinel seems more likely in the present case.

The calcined samples were investigated with STEM to obtain the initial size distribution of the NPs on the corresponding supports. Additionally, EDX mapping was used to identify the elemental composition of individual NPs. Particles supported on nanocrystalline SiO_2_ powder can be found nicely spread all over the support (Figure 2A). The average NP diameter extracted from the STEM images is 3.1 ± 0.8 nm for the particles supported on SiO_2_ (the particle size histogram is shown in Figure S2). On ZnOAl, the NPs cannot be easily distinguished from the support because of the low contrast between the elements involved (Cu, Zn, and Ga), with similar atomic numbers. Therefore, EDX mapping was used to identify the CuGa NPs on the ZnOAl support (Figure 2B). The EDX mapping for Cu looks very similar for both catalysts (Figure 2 E–H). However, the identification of the particles on the ZnOAl support is still challenging. The same applies to detecting Ga in the EDX maps due to its very low concentration within the NPs. It seems that Cu forms irregular particles, while Ga might be spread out over the whole ZnOAl support, although we are here within the uncertainty of the measurements for Ga.

**Figure 2:**
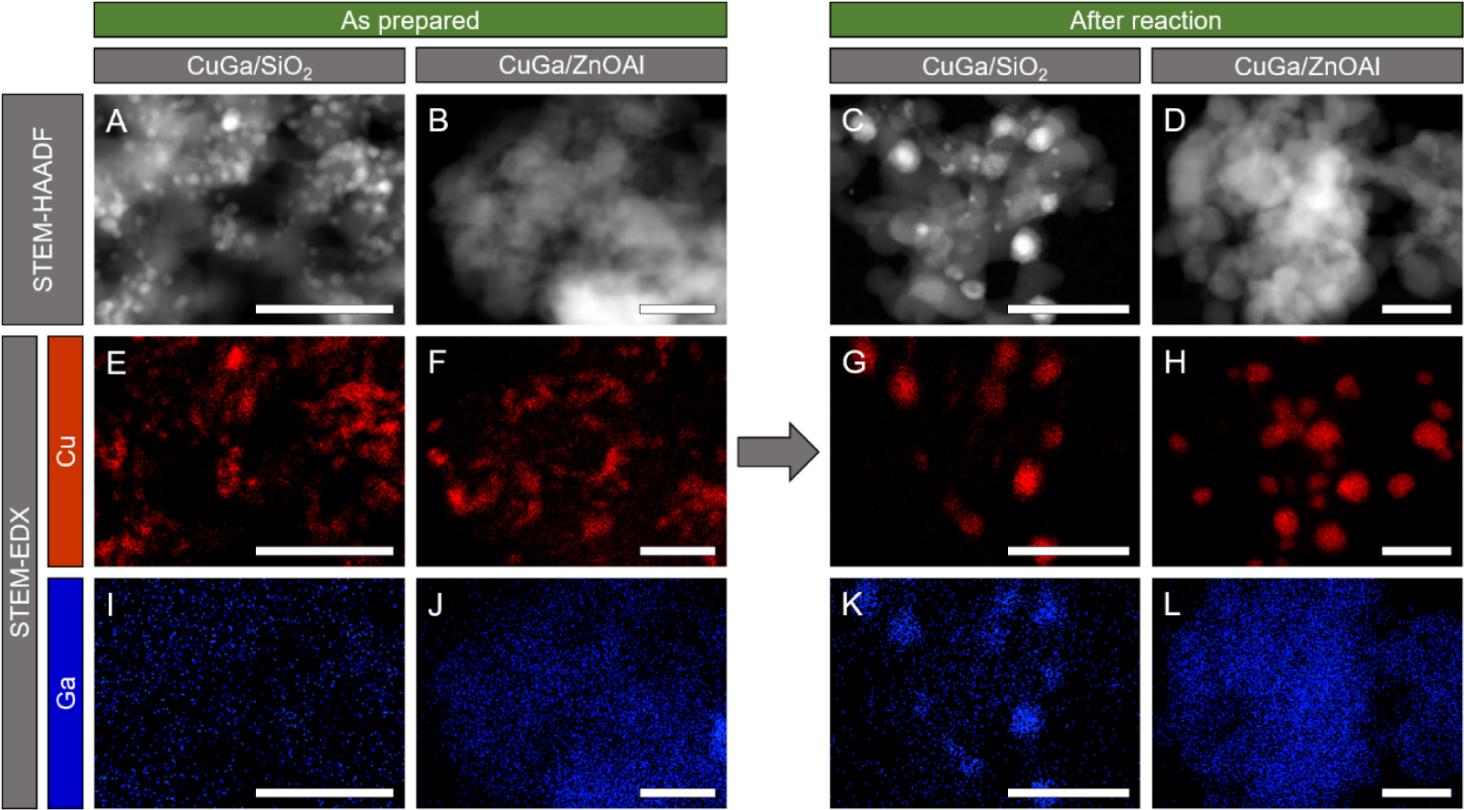
(A–D) STEM-HAADF images and corresponding EDX maps of (E–H) Cu and (I–L) Ga of CuGa NPs deposited on SiO_2_ and ZnOAl. The samples were measured as-prepared and after CO_2_ hydrogenation (H_2_ + CO_2_, 250 °C). The size of the scale bar in all images is 50 nm. The corresponding EDX spectra are shown in Figure S3.

To get more insight into the reaction-induced changes, the powder samples were also examined with STEM and EDX after the reaction (CO_2_ + H_2_, 250 °C). For both catalyst types, an increase of the average NP size could be observed in the course of the reaction. In addition, the NPs could be more easily identified on SiO_2_, where the average particle size after the reaction was 5.4 ± 4.3 nm. However, there is still a large number of smaller particles (≤5 nm) that correspond to the majority of the particles (>70%). Additionally, the average number of NPs in the images appears to have decreased (Figure 2C and D). This suggests that some of the NPs sinter and merge into larger ones. This behavior is most likely induced by the thermal pretreatment.

For the CuGa/SiO_2_ sample after reaction, the Cu and Ga contributions are found at the same locations in the EDX maps (Figure 2G and K). Therefore, Cu and Ga appear to be in close contact, and the NPs remain as bimetallic CuGa particles. Interestingly, this is not the case for the ZnO-containing catalyst after the reaction. Here, larger concentrations of Ga cannot be found associated with the Cu particles (Figure 2H and L). Instead, Ga appears dispersed over the whole sample, including the ZnOAl support. Locally, even clusters of Ga are found (Figure 3), but these are separated from the Cu particles. Therefore, Ga does not stay with Cu, but instead segregates from it and probably spreads over the ZnOAl support. This phenomenon could be explained by the different surface energies of SiO_2_ and ZnO.^51–53^ The lower surface energy of SiO_2_ will make its coating (wetting) by gallium (oxide) less favorable.

**Figure 3:**
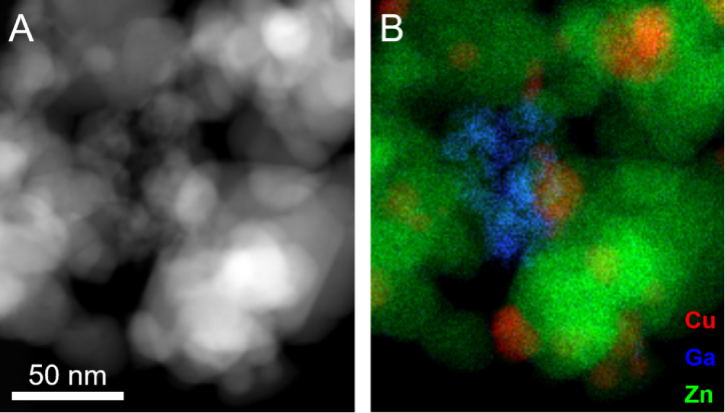
(A) STEM and (B) corresponding EDX map of the CuGa NP/ZnOAl catalyst after reaction (H_2_ + CO_2_, 250 °C). The corresponding EDX spectra are shown in Figure S4.

### Catalytic Performance

To measure the catalytic performance of the nanocrystalline powder catalysts, all samples were first reduced in H_2_ at 250 °C for 2 h to activate the catalyst. Next, the feed gas was changed to H_2_ and CO_2_ in a ratio of 3:1, and He was used as an internal standard.

First, it will be shown that there is indeed a promotional effect when adding Ga to a Cu catalyst even when deposited on an inert support such as SiO_2_. The CuGa/SiO_2_ catalyst is compared here to the Cu/SiO_2_ and CuZn/SiO_2_ catalysts that were used in a previous study.^40^ Details on the preparation and characterization of such catalysts can be found in ref 40, and the Cu:metal ratios employed are displayed in Table 1. It should be however noted that in contrast to the previous study, a feed gas mixture consisting of H_2_ + CO_2_ was used here for all measurements instead of H_2_ + CO_2_ + CO as in the previous study.

**Table 1:** Comparison of the ICP results for Cu, CuZn, and CuGa micellar NPs supported on SiO_2_

**Catalyst**	**Cu:M ratio** **(**M **= Zn, Ga)**	**Total metal loading (%)**
Cu/SiO_2_	-	2.60
CuZn/SiO_2_	1.65	2.23
CuGa/SiO_2_	8.67	2.90

The total NP loading with respect to the SiO_2_ support in all three catalysts is 2–3%, while the relative amount of Cu to the second metal (Zn or Ga) varies, with 62.3% Cu in the CuZn NPs and 89.6% Cu in the CuGa NPs. As can be seen from Figure 4, despite the low Ga content, the bimetallic CuGa NPs are superior to the pure Cu NPs, with an increase in the methanol yield by a factor of 8.4 when normalized by the total amount of Cu in the catalyst. Moreover, even though a lower Cu:M content is available in the CuZn NPs as compared to CuGa, an increase in the methanol yield by almost a factor of 25 is obtained versus the pure Cu NPs. In addition, all catalysts also produce a small amount of methane as a side product. The amount produced is similar for all catalysts, but because of the higher methanol production for the CuGa and CuZn NPs, in such samples, CH_4_ makes an overall lower fraction of the selectivity.

**Figure 4:**
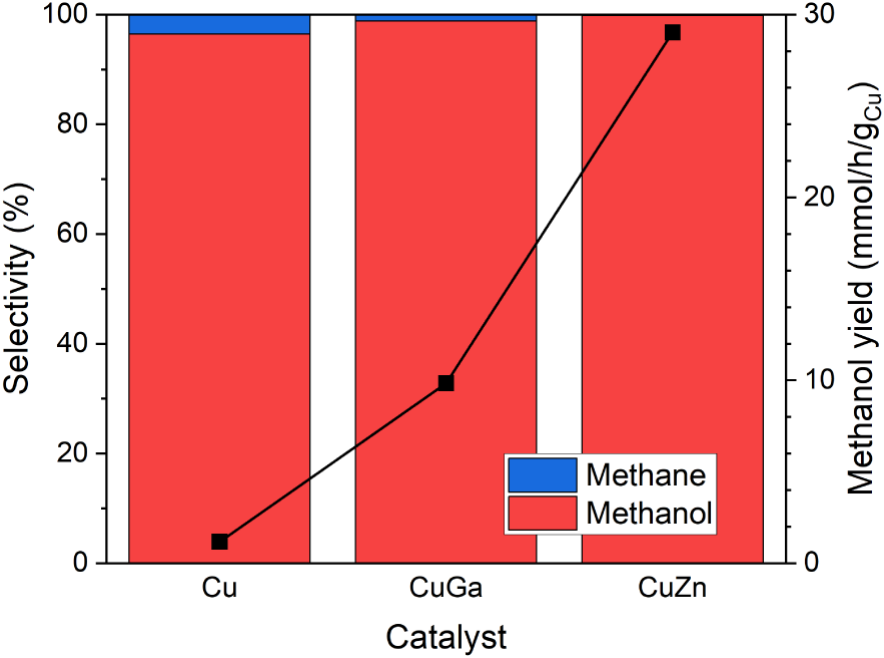
Comparison of bimetallic micellar Cu_90_Ga_10_, Cu_62_Zn_38_ and pure Cu NPs, all supported on nanocrystalline SiO_2_. The reactivity was measured at 40 bar and 250 °C in a feed gas mixture of 60% H_2_, 20% CO_2_, and 20% He. The methanol yield is normalized to the total amount of Cu in the catalyst. The flow rate for these measurements was 17 mL/min. The data points were obtained at 40 bar and 250 °C. The contribution of CO is excluded from the selectivity plot.

Similar to the case of model micellar CuZn NPs investigated previously by our group,^40^ the activity (methanol yield) of the micellar CuGa NPs is inferior to that of the commercial catalyst. There are however a number of distinctions among the model and the performance catalysts that can result in such differences, including a drastically lower Cu loading, distinct average initial particle structures (size and shape), support structure, and composition. Thus, the goal of our investigations targeting the model system presented is not to unveil the world’s most active catalyst but to get deeper insight into the Cu–Ga interaction. This can only be accomplished when one is able to change a parameter at a time, i.e., by decreasing the complexity of the materials system already in the precatalyst state. This is achieved here by keeping a similar initial particle size, dispersion, and surface composition. Moreover, despite the clearly lower activity, the methanol selectivity of the CuGa micellar particles is still very high (close to 100%, when excluding CO produced by the RWGS).

To get more information about the effect of Ga on the catalyst, the next section will focus on the CuGa NPs supported on either SiO_2_ or ZnO. The reactivity of the catalysts was then measured at atmospheric pressure as well as at 20, 40, and 60 bar. The catalytic performance was normalized to the amount of Cu in the sample, which was determined by ICP. The amount of Cu and Ga in the catalysts obtained from ICP is shown in Table S1.

As presented in Figure 5A, the catalytic performance of the ZnOAl-supported catalyst is much better than that of the SiO_2_-supported catalyst. Not only is it more active (about 5–9 times higher CO_2_ conversion depending on the pressure), but it is also more selective. At 40 bar, the selectivity toward methanol for the CuGa/SiO_2_ catalyst is 23.5%. In contrast, the selectivity of the CuGa/ZnOAl catalyst under the same conditions is ≈58%. For both catalysts, the main products are either methanol or CO produced by the reverse water gas shift (RWGS) reaction. Both catalysts also produce minor amounts of CH_4_, although the maximum product selectivity toward methane is below 0.7% in all measurements (Figure 5B). The importance of the Cu–ZnO synergy for methanol synthesis is well-known. SiO_2_ in contrast does not greatly influence the activity of Cu catalysts.^5,54^ The much higher activity observed for the CuGa/ZnOAl catalyst may therefore mainly originate from active sites at the Cu–ZnO interface and not from Cu–Ga or Zn–Ga interactions. Nonetheless, Ga_2_O_3_ was also shown to improve the performance of Cu catalysts, although not as much as ZnO.^5^ This is in line with our observations here, where the CuGa/SiO_2_ catalyst is less active and selective than the CuGa/ZnOAl catalyst.

**Figure 5:**
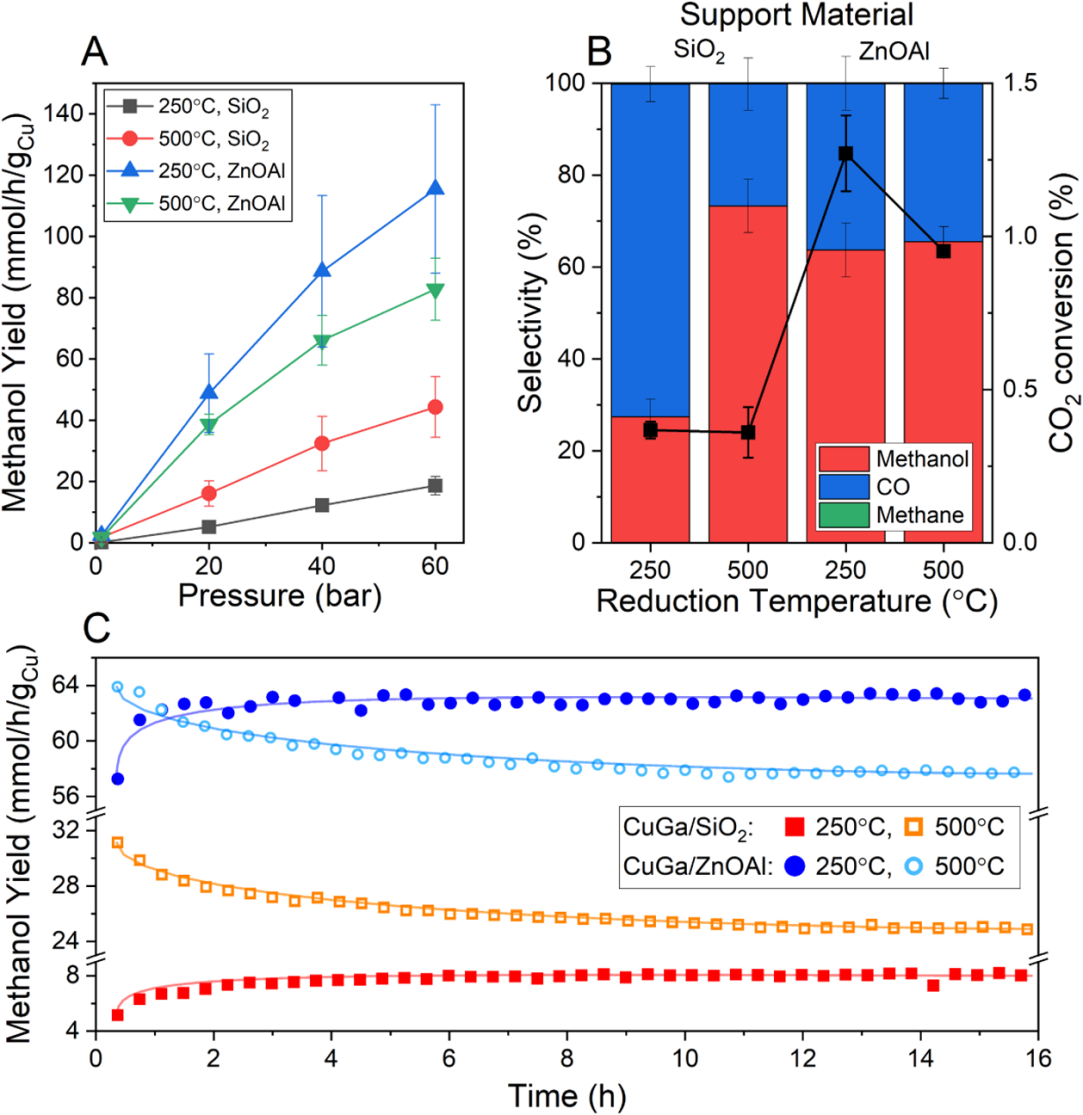
(A) Methanol yield and (B) product selectivity and CO_2_ conversion of CuGa/SiO_2_ and CuGa/ZnOAl catalysts. The product selectivity and the CO_2_ conversion in (B) are shown only at 40 bar. (C) Temporal evolution of the methanol yield after reduction pretreatments at 250 and 500 °C. All reactivity measurements were conducted at a reaction temperature of 250 °C. Lines in (B) and (C) serve as a guide for the eye.

We assume that Ga species stay oxidized under these reaction conditions (H_2_ + CO_2_, 250 °C). This assertion will be later supported by our XAS measurements. In order to attempt a further Ga reduction, a second annealing treatment in 10% H_2_ (balanced in He at atmospheric pressure) was undertaken, but this time, the temperature was increased to 500 °C. After 2 h, the reactor was cooled to 250 °C (the same temperature as in the previous experiment), and the reactivity measurements of the sample were repeated. The high temperatures used during this reduction pretreatment were expected to reduce Ga in the sample. At the low Ga:Cu ratios (≈10%) used here, the potential alloy that will form is a solid solution with the fcc structure of pure Cu, but modified by replacing Cu atoms with Ga.^30^

As shown in Figure 5, both catalysts react quite differently to the high-temperature treatment. The NPs supported on SiO_2_ show a higher methanol yield after the 500 °C pretreatment. For this catalyst, the methanol yield is in fact more than double at all pressures, although it is more pronounced at the lower pressures, with a nearly three times higher methanol yield at 20 bar. Interestingly, the total CO_2_ conversion of this catalyst did not increase (Figure 5B). Instead, the selectivity significantly shifted from CO (produced by the RWGS) to methanol (produced by CO_2_ hydrogenation). Thus, this 500 °C thermal pretreatment resulted in the creation of new or modified active sites on the CuGa/SiO_2_ catalysts for the methanol synthesis reaction. These new sites may also contain reduced metallic Ga or Cu–Ga alloys, which are only formed at the higher reduction temperatures.

In contrast, for the CuGa/ZnOAl catalyst, a slight deactivation is observed (Figure 5B). For this sample, the decrease in the methanol yield becomes more pronounced at e higher pressures, with a drop in the methanol yield of 1.8%, 9.3%, and 17.4% at 20, 40, and 60 bar, respectively. This drop is also linked to a lower conversion rate of CO_2_. The deactivation could be explained by sintering of the particles and loss of surface area, which is likely to occur at such high temperatures and was also confirmed by STEM (Figure 2). It is also plausible that, due to the different mixing energies, Zn atoms replace the Ga atoms, and the possible Cu–Ga alloy might turn into a Cu–Zn (brass) alloy.^30^ Therefore, on the CuGa/ZnOAl sample, the majority of the activity appears to still come from the Cu–ZnO interaction. This means that brass formation at high temperatures is also a possible explanation for the decreased activity of the CuGa/ZnOAl catalyst since Cu–Zn alloys have been reported to be less active for methanol synthesis than Cu/ZnO.^36,55^ Furthermore, Ga and Zn can potentially form a spinel structure,^18^ and due to the abundance of Zn in the ZnO-supported catalyst, no more Ga might be available at the sample surface to interact with the Cu in the NPs. Instead, Ga might be incorporated as Ga^3+^ in ZnO, as it was also observed before.^35^

For additional insight, the changes in catalytic activity with time on stream after the different reduction pretreatments were also analyzed, Figure 5C. For this, the reactivity was measured in the H_2_ + CO_2_ mixture at 250 °C and 40 bar, directly after the reduction. As shown in Figure 5C, for both 500 °C pretreated catalysts, the activity decreases before converging at a stable value after 12 h. In contrast, the steady state was achieved much faster (ca. after 2 h) for the 250 °C pretreated samples. The absolute decrease of the methanol yield for the 500 °C pretreated catalysts was very similar for both catalysts, about 6.2 mmol/h/g_Cu_. A loss of activity due to sintering could explain these findings.

Interestingly, after the reduction treatment at high temperature, the selectivity of the CuGa/SiO_2_ catalyst was significantly improved. However, the relative loss of activity over time, when going back to the original 250 °C reaction conditions, is a bit higher for this catalyst. A reason for this may be a (partial) reversal of the effects of the high-temperature treatment, specifically the loss of the modified methanol-selective active Cu–Ga sites. To link the observed changes to the actual changes in the structure and chemical state of the catalyst, in situ spectroscopic characterization was applied.

### In Situ Structure/Composition Characterization (XAS and NAP-XPS)

The effect of the Cu–Ga interaction is much more pronounced on the SiO_2_-supported NPs. Therefore, to gain insight into the changes happening under reaction conditions, the CuGa NPs supported on SiO_2_ were studied with XAS. All XAS measurements were performed at atmospheric pressure. The results of these measurements are shown in Figure 6. By comparing the edge steps (absorption jumps) at the Cu and Ga K-edges of spectra measured in the transmission mode, one can estimate the elemental composition of the sample. For the measured sample, the edge steps were Δ­(μd)_Cu_ = 0.52 and Δ­(μd)_Ga_ = 0.05, which match the 10:1 ratio determined by ICP, thus confirming the previously measured elemental composition.

**Figure 6:**
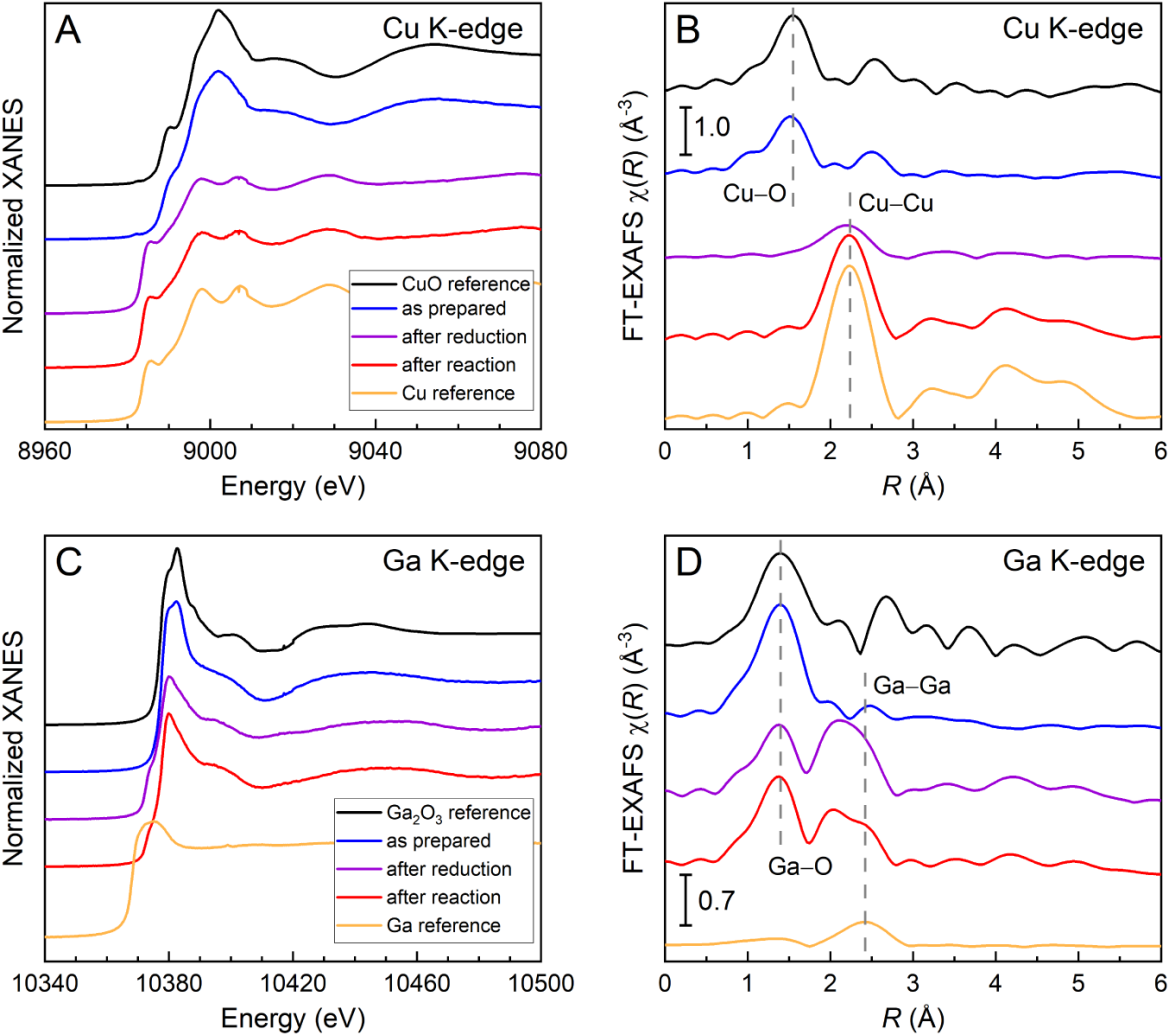
Selected (A, B) Cu K-edge and (C, D) Ga K-edge XANES and Fourier-transformed EXAFS spectra of the CuGa/SiO_2_ catalyst in the as-prepared state, after reduction (H_2_, 500 °C) and after reaction (H_2_ + CO_2_, 250 °C). Additionally, reference spectra of CuO, metallic Cu, Ga_2_O_3_, and metallic Ga are shown. The reference lines in (B) indicate the peak positions of the Cu–O and Cu–Cu bonds obtained from the CuO and metallic Cu references, respectively. The reference lines in (D) indicate the peak positions of the Ga–O and Ga–Ga bonds obtained from the Ga_2_O_3_ and metallic Ga references, respectively.

In their initial state, the CuGa NPs are fully oxidized, as expected after the calcination treatment in O_2_ for ligand removal. As it can be seen from the comparison of X-ray absorption near-edge structure (XANES) and Fourier-transformed extended X-ray absorption fine structure (EXAFS) spectra for the CuGa catalyst with those for the reference compounds, copper is oxidized to the 2+ state (Figure 6A) and gallium is oxidized to the 3+ state (Figure 6C). However, there are small differences between the XANES spectra from the sample and the reference materials. We attribute these differences in the relative intensity of the features to a higher degree of structural disorder in the small CuGa NPs as compared to the bulk Ga_2_O_3_ reference. This is also backed up by the EXAFS data. The Fourier-transformed (FT)-EXAFS spectrum at the Ga K-edge is dominated by the contribution of the first coordination shell (corresponding to Ga–O bonds) and does not show significant contributions from the more distant coordination shells. This suggests a strongly disordered, amorphous structure of Ga oxide, which is also in agreement with the XRD characterization (Figure S1). The β-Ga_2_O_3_ modification is the most stable gallium oxide structure, where the Ga ions are either 4- or 6-fold coordinated in tetrahedral and octahedral sites, respectively. Using this structure as a starting parameter for the fit of the Ga K-edge EXAFS spectrum of the as-prepared catalyst gives a coordination number (CN) of 3.8 for the Ga–O bond (i.e., lower than the average Ga–O CN of 5 in the Ga_2_O_3_ structure), which also points to a strongly disordered environment of the Ga species or to the nanosized character of the particles. The initial Ga K-edge spectra can be well fitted by just using a first shell Ga–O scattering path. The best fit parameters for the Ga K-edge EXAFS can be found in Table S2. The behavior of the sample under mildly reducing conditions (H_2_ atmosphere at 250 °C) is investigated in Figures 6 and 7. Under these conditions, the copper oxide (CuO) gets completely reduced to metallic copper, as previously observed for similarly prepared pure Cu and bimetallic CuZn particles.^40^ Gallium on the other hand stays oxidized (Ga^3+^), and no major changes are observed.

**Figure 7:**
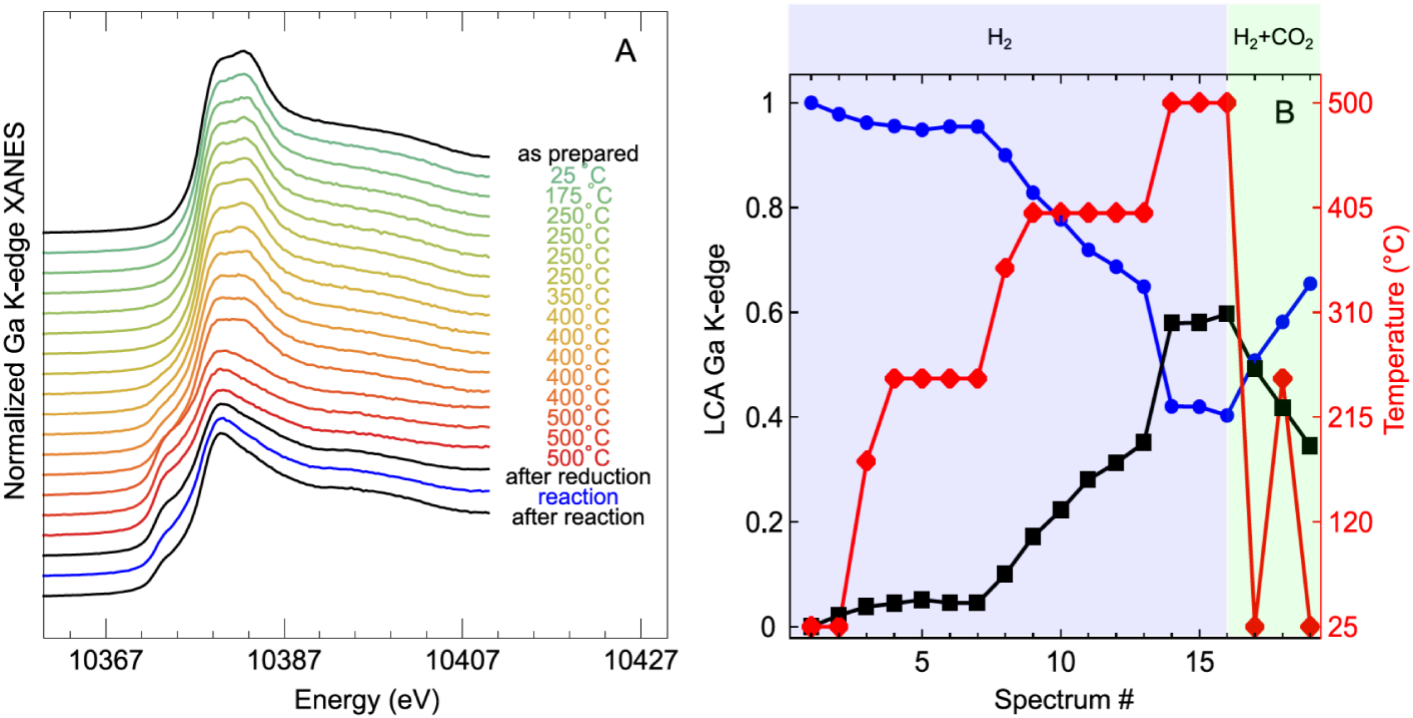
Linear combination analysis (LCA) of Ga K-edge spectra in CuGa NPs/SiO_2_. (A) In-situ XANES spectra of the Ga K-edge that were used for LCA. (B) Evolution of the fractions of metallic Ga (black squares) and oxidized Ga (blue circles) under different H_2_ and CO_2_ + H_2_ treatments at various temperatures (all at 1 bar) as obtained from the LCA using reference spectra.

Clear changes in the catalytic performance were seen for higher reduction temperatures (Figure 5). Therefore, in the next steps, the temperature was stepwise increased, first to 400 °C and then to 500 °C. At these temperatures, a partial reduction of gallium is observed. This is indicated by a reduction of the intensity of the main peak in the Ga XANES spectra and the formation of a shoulder at the onset of the absorption edge, but the spectrum is still different from that of metallic Ga, which suggests that Ga is not fully reduced (Figure 7). This behavior is also confirmed by the EXAFS spectra. A clear contribution corresponding to Ga–M bonds (here M is Ga or Cu) appears at 500 °C. Still, the contribution of Ga–O remains clearly visible in the EXAFS spectra, indicating only partial reduction of Ga. As copper is already fully reduced at 250 °C, no changes are observed for the Cu K-edge at higher temperatures, except those related to an increase in thermal disorder.

After the reduction in H_2_, the sample was cooled to room temperature in the same gas atmosphere to collect high-quality EXAFS spectra that are not dampened by thermal disorder. The quantitative analysis of the partially reduced CuGa structure was performed by fitting these Ga K-edge EXAFS spectra (Table S2). In agreement with the visual examination of the XAS data, a Ga–M (M = Cu, Ga) component had to be included in the EXAFS fitting. The bond length of this feature is too short to be a higher coordination shell from Ga_2_O_3_, but instead, it is assigned to a metallic contribution that appears under these conditions. Here, it is plausible that some Ga atoms get integrated in the fcc-Cu lattice and a Cu-rich CuGa alloy forms as it would be expected from the phase diagram.^30^ The coordination number of this bond Ga–M is 4.3, but there is still a Ga–O contribution with a CN of 2.0. This shows that gallium is only partially reduced.

The results obtained from the EXAFS analysis of the samples also match those from the linear combination analysis (LCA) of the XANES data of the Ga edge (Figures 6 and S5). The LCA shows an increase in the fraction of metallic Ga already at 400 °C, which seems to stabilize at below 40%. However, at 500 °C, this amount of reduced Ga is further increased. We acknowledge here that LCA using the XANES spectrum of metallic Ga as a reference does not result in a perfect fit for our in situ XANES data, especially at higher temperatures. We attribute this discrepancy to the fact that the final state of the reduced Ga in CuGa alloys is quite different from the structure of the metallic Ga reference (the former has a fcc structure and much shorter metal–metal distance, which is visible also from the position of post-edge features in our in situ XANES spectra).

Following the reduction treatment, the gas mixture in the chamber was changed to 25% CO_2_ and 75% H_2_, and the temperature was increased to 250 °C, matching the reaction conditions used in our catalytic studies. The spectra show that the Cu component stays reduced, but Ga is partially reoxidized under the reaction conditions (Figures 6 and 7). This can also be seen in the results of the quantitative fitting of the EXAFS spectra, which were performed after the sample was cooled to room temperature to minimize the effect of thermal disorder. These spectra showed a decrease in the Ga–M CN, together with a simultaneous increase of the Ga–O CN (Table S2), unveiling sample reoxidation. The reoxidation is only partial because there is still a metallic contribution visible in the XANES and EXAFS spectra. Similar effects were seen in a model study of Ga deposited on Cu(100), where it was observed that even minimal amounts of oxygen can oxidize Ga, which will then remain stable in its oxidized state even under reducing reaction conditions (CO_2_ + H_2_, 1 bar).^56^

Our results show that even if Ga is successfully (partially) reduced in H_2_ at high temperatures (>400 °C) during pretreatment, metallic Ga is not stable under the applied reaction conditions (CO_2_ + H_2_, 250 °C). Instead, partial reoxidation occurs in the reaction mixture. Furthermore, the results obtained from the XAS measurements can be linked to the prior reactivity data. In particular, the increased methanol selectivity obtained after high-temperature sample pretreatment for CuGa/SiO_2_ can be linked to the partial reduction of Ga_2_O_3_ and dilute Cu–Ga alloy formation. Nonetheless, this configuration is again partially modified under reaction conditions (dealloying and formation of Ga_2_O_3_), which might explain the time-dependent decrease in the methanol yield that was detected. Note that the deactivation (as shown in Figure 5C) will not match the amount of oxidation exactly because pure Cu or a mix of Cu and Ga_2_O_3_ (not present as an alloy) are also active catalysts (as seen in our previous comparison, Figure 4). Since having reduced Ga leads to a more active catalyst, the activity should decrease if the catalyst is oxidized again. Nonetheless, it appears that even after 12 h operation, some of the formed metallic Ga still remains since a better methanol selectivity is still obtained on the samples pre-exposed to the high-temperature pretreatment.

It seems that Ga in this catalyst, similarly to Zn in a Cu/ZnO catalyst, can be reduced during a pretreatment in H_2_, which will then affect the catalytic performance of the system. This reduction can be (partially) reversed again under reaction conditions in both cases.^37^ Another similarity is that the oxidation state, and thus the promotional effect, can be tuned by the amount of Ga^19,34^ or Zn.^36,57^ However, it should be noted that GaO_*x*_ species become reduced at higher temperatures than ZnO. Also, while the reduction of ZnO to metallic Zn and thus the formation of a CuZn alloy are seemingly detrimental for the catalytic performance,^40^ the same is not true in the case of Ga. To the contrary, our measurements show a higher activity for CuGa/SiO_2_ when compared to (Cu + GaO_*x*_)/SiO_2_ which was available upon the sample pretreatment at lower temperature.

Finally, since XAS is sensitive to all absorbing atoms of a given element in the sample, it can be difficult to obtain information from the surface of the NPs, where the reactions are happening. In fact, even though the NPs in the present study were relatively small (∼3 nm) and a significant number of atoms are present at the particle’s surface, the signal arising from the atoms within the bulk of the particle is still expected to dominate the overall XAS data recorded here. To obtain surface-sensitive information about the structure of the CuGa NPs during the reaction and to understand the differences in their interaction with the ZnO and SiO_2_ support materials, NAP-XPS experiments were conducted. Here, the Cu_70_Ga_30_ NPs were supported on planar SiO_2_/Si­(111) and ZnO(0001) substrates. To achieve a good Ga signal under *in situ* conditions for these experiments, the Ga content in these particles had to be slightly increased (70:30) as compared to the NPs in our powder-supported catalysts (ca. 90:10).

The NAP-XPS spectra were collected at two different photon energies to obtain depth-sensitive information on the NPs. The peak fitting results of the spectra are shown in Figures 8, S6 and S7 for the NPs supported on SiO_2_/Si and ZnO. The first step of the experiment was to look at the samples under ultrahigh vacuum (UHV) conditions. The only treatment that the samples experienced before was O_2_ plasma cleaning to remove the encapsulating polymer from the NP synthesis. The initial elemental composition of the NPs is close to the nominal Cu:Ga ratio of 70:30, but overall, a bit more Ga than expected is found in the samples in their initial state in UHV (Cu:Ga ratio is ≈60:40). The evolution of the calculated atomic fractions of Cu and Ga in the NPs during all steps of the experiment is shown in Figure 8C and F. The mismatch in the initial Cu:Ga ratio may be explained by an already small effect on the NP structure by the pretreatment in atomic oxygen. Both metals are initially oxidized, although the Cu spectra still showed a contribution from Cu^+^/Cu^0^ in addition to the dominant 2+ state (Figures 8, S6 and S7).

**Figure 8:**
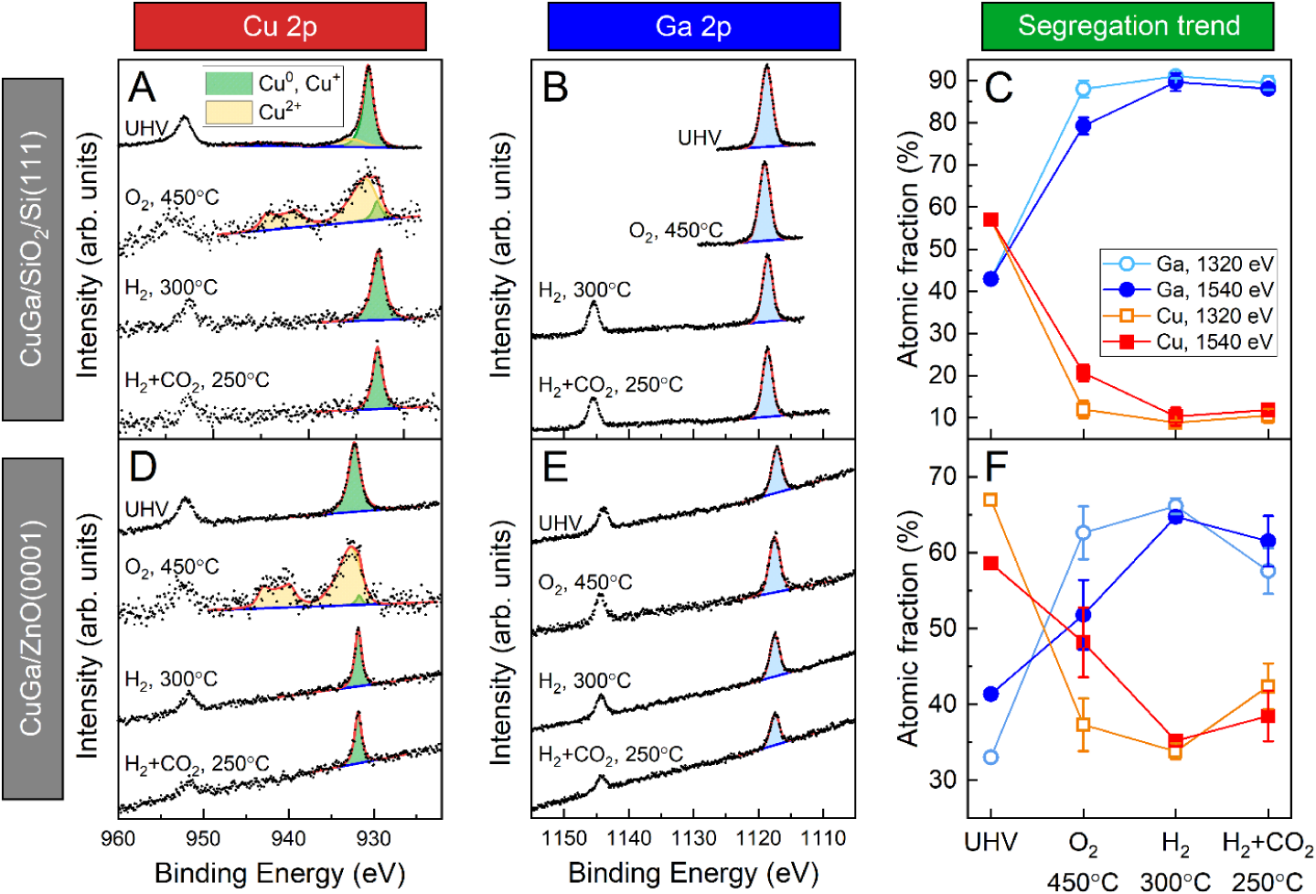
Representative NAP-XPS spectra of the (A, D) Cu 2p and (B, E) Ga 2p regions for the Cu_70_Ga_30_ NPs supported on SiO_2_/Si­(111) and ZnO(0001) measured with a photon energy of 1540 eV. The spectra shown here were acquired under ultrahigh vacuum (UHV), oxidizing (450 °C, O_2_, 0.9 mbar), reducing (300 °C, H_2_, 1 mbar), and reaction conditions (250 °C, H_2_ + CO_2_ (3:1), 1 mbar). The relative atomic fractions of Cu and Ga, acquired for two different probing depths, show segregation trends for the NPs supported on (C) SiO_2_/Si­(111) and (F) ZnO(0001). These values were calculated from the 2p_3/2_ peak areas of the corresponding spectra, and only the contributions from Cu and Ga were included.

In order to remove the adventitious carbon present on the sample surface due to the *ex situ* sample transfer to the synchrotron facility, an *in situ* annealing treatment at 450 °C in O_2_ (0.9 mbar) was applied. This high temperature is also used here to mimic the calcination treatment for the powder catalysts. The C 1s region was monitored during this process, and the treatment was continued until no carbon signal could be detected from the sample surface. During this process, further oxidation of Cu was observed. The oxidation state is now mostly Cu^2+^, although a small contribution of Cu^0^/Cu^+^ still remains (Table S3). Interestingly, the concentration of Cu^2+^ is higher for the higher photon energy of 1540 eV, meaning that the sample is less oxidized at the surface, but only for the NPs supported on ZnO. As described above, already from the beginning, there seems to be a mixture of multiple components contributing to the Cu spectra. The presence of Cu^2+^ can be clearly identified by a strong shakeup satellite, but there is at least one additional component, probably metallic Cu^0^ or Cu^+^ oxide. These two contributions are hard to distinguish because of their similar binding energy values. Usually, one can use the shape of the Cu LMM Auger contribution to identify Cu^0^ or Cu^+^. Unfortunately, due to the low intensity of the signals for this submonolayer bimetallic NP coverage on the support surface, the Auger regions could not be reliably recorded. Because of the *ex situ* O_2_ plasma treatment, one can only assume that the initial spectra likely correspond to a mixture of Cu^+^ and Cu^2+^ oxides.

The oxidation state of Ga is identified by the position of the Ga 2p peak. Metallic Ga has a binding energy of 1116.49 eV, and the position for Ga_2_O_3_ is at 1117.80 eV, which makes these two species clearly distinguishable by their binding energy.^46^ Over the course of the whole experiment, the peak position does not significantly change. Therefore, one can assign the peaks to Ga_2_O_3_, meaning that Ga stays oxidized during the whole experiment.

Additionally, a change of the elemental surface composition during the oxidation treatment can be observed for the NPs. The signal for Ga increases, while the one for Cu decreases (Figure 8). Because of the surface sensitivity of XPS, this can be interpreted as a surface segregation of Ga. In this case, Ga would be located at the surface and Cu in the core of the NPs. This trend is more pronounced for the particles on the SiO_2_/Si­(111) substrate. At a photon energy of 1320 eV for the SiO_2_-supported sample, the Ga amount is 88%, while for the ZnO sample, it is only 63%. At the higher photon energy (1540 eV), the Ga fraction is a bit lower, with 79% and 52% for the particles on SiO_2_ and ZnO, respectively. This fits well with a gallium enrichment at the surface.

In the next step, the chamber was filled with H_2_ (1.0 mbar) and heated to 300 °C to reduce the NPs. Note that a slightly higher temperature is chosen here for the reduction as compared to the other experiments. This is to ensure a reduction of the copper phase similar to the XAS and reactivity measurements because the complete reduction of Cu at low pressures only happens at higher temperatures.^58^ Under these conditions, the complete reduction of Cu can be indeed observed with NAP-XPS. Only one peak originating from Cu^0^ or Cu^+^ remains. From the experience and former experiments with similar bimetallic NPs (CuZn^40^ or CuNi),^38^ it is fair to assume that Cu is most likely fully reduced to Cu^0^ and no Cu^+^ remains under these conditions. The Ga component, nevertheless, remains unchanged (Ga_2_O_3_) in agreement with the XAS results for the powder-supported catalyst.

Furthermore, a more pronounced reorganization of the metals in the NPs can be observed. Specifically, the Ga segregation to the surface of the particles is even stronger, as indicated by an increase of the Ga/Cu ratio in NAP-XPS. This trend is similar to the one observed before for Cu–ZnO particles,^2,40^ where ZnO segregates to the surface under reducing conditions. Interestingly, the values obtained for the Ga/Cu ratios are similar for both photon energies. This suggests the formation of an equilibrium concentration at the surface, which consists of not only mainly Ga_2_O_3_ but also low amounts of Cu. Nonetheless, the surface segregation trends appear different for the distinct supports chosen, despite the use of the same NP solution (same initial Cu/Ga ratio in the precatalyst NPs). While the Ga surface concentration is around 65–66% for the NPs on ZnO, it is 90–91% for the SiO_2_-supported NPs. The higher abundance of Cu at the surface of the CuGa/ZnO sample suggests a stronger interaction between the particles and the oxide support.

The interaction of the particles on the different supports is schematically represented in Figure 9. This stronger interaction of the NPs with ZnO can result in flatter CuGa NPs^59,60^ and/or the incorporation of Ga into the ZnO support.^35^ However, Ga species probably remain in their 3+ state because no signal of reduced Ga is seen. For Cu NPs on ZnO, this strong interaction between the NPs and the reducible oxide support (SMSI) is well-known. It was also shown that the wetting/dewetting behavior of pure Cu is different on SiO_2_ and ZnO.^61^ We propose that this is also the case for CuGa NPs, with a stronger wetting of the ZnO than of SiO_2_. This makes sense due to the much higher surface energy of ZnO as compared to SiO_2_.^51–53^ This results in flatter particles with higher surface-to-volume ratios. Therefore, the surface layer of Ga becomes thinner, and a higher amount of Cu is closer to the surface of the particles, which explains the observed Ga/Cu ratios. An additional factor could be the migration of Ga from the NPs to the support. This would have a similar effect in terms of the observed Ga/Cu ratios. Furthermore, the migration of Ga away from the Cu and the formation of a Ga cluster were already observed for the powder catalyst after reaction by our STEM measurements (Figure 2).

**Figure 9:**
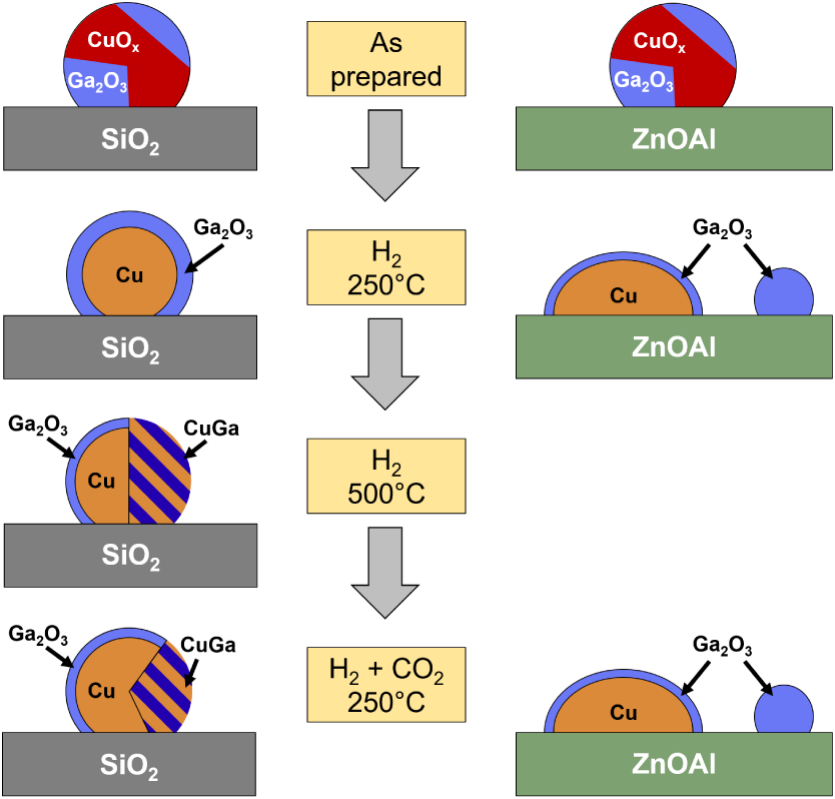
Schematic representation of the proposed evolution of the CuGa NPs and their interaction with the support of the different materials upon exposure to different thermal treatments and chemical environments.

In the next step, the sample was exposed to the reaction conditions. To duplicate the conditions from the other experiments, a 3:1 mixture of H_2_ and CO_2_ (1.0 mbar total) was used, and the temperature was set to 250 °C. Unfortunately, due to limitations in the experimental technique, a pressure gap between this experiment and the catalytic measurements remains. No further significant change in the Cu:Ga ratios is observed for the NPs on SiO_2_. However, for the samples on ZnO, a slight reversal of the previously observed trend can be seen, resulting in a slight enrichment of the surface with Cu, even though the surface still stays gallium-rich. No changes in the oxidation states of either Ga or Cu are detected during this step.

In summary, Ga-surface segregation was observed for the NPs deposited on both oxide supports, although the effect is stronger on CuGa/SiO_2_ as compared to CuGa/ZnO. An explanation for this can be derived from the different interaction of the NPs with the oxide support due to their distinct surface energies, Figure 9. The stronger particle-support interaction on ZnO would lead to stronger wetting and, therefore, flatter particles with more Cu located closer to the surface, which can be more easily detected with XPS. This may also explain the higher activity of the CuGa/ZnO system since it implies that the creation of Cu–ZnO sites is responsible for the majority of the activity in this sample. An alternative reason can be the migration of Ga from the CuGa particles to the ZnO surface, where it might be present as GaO_*x*_ clusters. This would also match the STEM observations of the powder samples because the thinner Ga shell does not block the signal from Cu in the nanoparticles as strongly, and thus, a higher Cu amount can be detected by XPS. A combination of both processes presented here is also plausible.

## Conclusion

In this study, bimetallic CuGa NPs supported on either SiO_2_ or ZnO were investigated with various synergistic experimental approaches to get new insights into the nature of the promotional effect caused by the addition of Ga to Cu-based methanol synthesis catalysts. Our experiments show that Ga can improve the methanol yield and selectivity in CO_2_ hydrogenation; however, its promotional effect varies depending on the support material and pretreatment.

Cu and Ga only weakly interact with the SiO_2_ support, which enhances their interaction with each other and helps them to stay in close contact during the CO_2_ hydrogenation reaction. On the other hand, on the ZnO support, Ga appears to separate from Cu in the NPs and migrates over the ZnO support. Additionally, ZnO as a support or as part of the NPs already greatly increases the catalytic performance of the Cu particles by the Cu–ZnO synergy such that the added Ga does not lead to any major improvement. Therefore, the promotional effect of Ga is more significant for the particles supported on SiO_2_ as compared to those supported on ZnO.

Furthermore, pretreatments in H_2_ at different annealing temperatures (250 °C vs 500 °C), leading to distinct Cu–Ga interactions, were found to strongly influence the subsequent catalytic performance, especially of the CuGa/SiO_2_ catalyst, where the selectivity toward methanol is increased, while the rate of CO_2_ conversion remains constant. XAS revealed the presence of (partially) reduced Ga after this high-temperature treatment, which can be held responsible for the increased methanol selectivity of the high-temperature alloy phases present in the 500 °C annealed CuGa NPs/SiO_2_. However, under CO_2_ hydrogenation conditions, Ga (partially) reoxidizes, and this partially reverts the effect of the pretreatment. The catalyst eventually reaches a steady state, where Ga^3+^ and Ga^0^ species, potentially present as Ga species incorporated in the Cu structure, coexist. Thus, the higher selectivity toward methanol originates from the formation of a Cu–Ga alloy, which is originally created at high temperatures and then (partially) persists under reaction conditions. Dealloying that takes place in this case as a function of increasing reaction time was found to lead to catalyst deactivation. However, it should be noted that the addition of metallic Ga as well as Ga_2_O_3_ to a Cu catalyst does lead to an increased activity toward methanol. The promotional effect of metallic Ga or a CuGa alloy is just better at promoting this catalyst than just adding Ga_2_O_3_.

To summarize, this study illustrates that the catalytic performance of Ga-promoted Cu catalysts is dependent on the content and chemical state of the Ga promoter. This can be however to some extent controlled by the temperature and duration of the catalyst pretreatment. Moreover, the choice of the support material is also critical to achieve the promotional effect of Ga, including its role of keeping Cu and Ga in intimate contact. Thus, although bimetallic CuGa NPs are suitable catalysts for CO_2_ hydrogenation to methanol, the preparation and pretreatment or activation of the catalyst, as well as the choice of oxide support, have to be carefully chosen to maximize their performance and stability.

## Supplementary Material



## Notes

XRD patterns for catalysts and reference patterns; particle size histograms extracted from TEM images; EDX spectra of corresponding STEM-EDX measurements; spectra with corresponding fits of XAS LCA; representative NAP-XPS spectra of Cu 2p and Ga 2p regions (Figures S1–S7); atomic fractions of Cu and Ga in the catalysts obtained from ICP-MS; EXAFS best-fit parameters for the Ga K-edge; and XPS fit parameters for the catalysts at various reaction conditions (Tables S1–S3) (PDF)
