# Supplementary material for: Cu–Ga Interactions and Support Effects in CO2 Hydrogenation to Methanol Catalyzed by Size-Controlled CuGa Nanoparticles Deposited on SiO2 and ZnO
Source: ACS Catal. 2025 Oct 2;15(20):17241–54. doi: 10.1021/acscatal.5c03414 (PMC12538547; doi:10.1021/acscatal.5c03414)
Supplement: Supplementary file 1 [file cs5c03414_si_001.pdf]

Supplementary Information for

Cu-Ga Interactions and Support Effects in CO_2 Hydrogenation to Methanol catalyzed by Size-Controlled CuGa Nanoparticles deposited on SiO_2 and ZnO

David Kordus[‡], *Janis Timoshenko*[‡], *Núria J. Divins*[†], *See Wee Chee*[‡], *Eduardo Ortega*[‡], *Mauricio Lopez Luna*[‡], *Uta Hejral*[‡], *Ane Etxebarria*[‡] *and Beatriz Roldan Cuenya*[‡] *

[‡] Department of Interface Science, Fritz-Haber Institute of the Max Planck Society, Berlin 14195, Germany

[†] Department of Physics, Ruhr University Bochum, 44801 Bochum, Germany

* Corresponding author: roldan@fhi-berlin.mpg.de

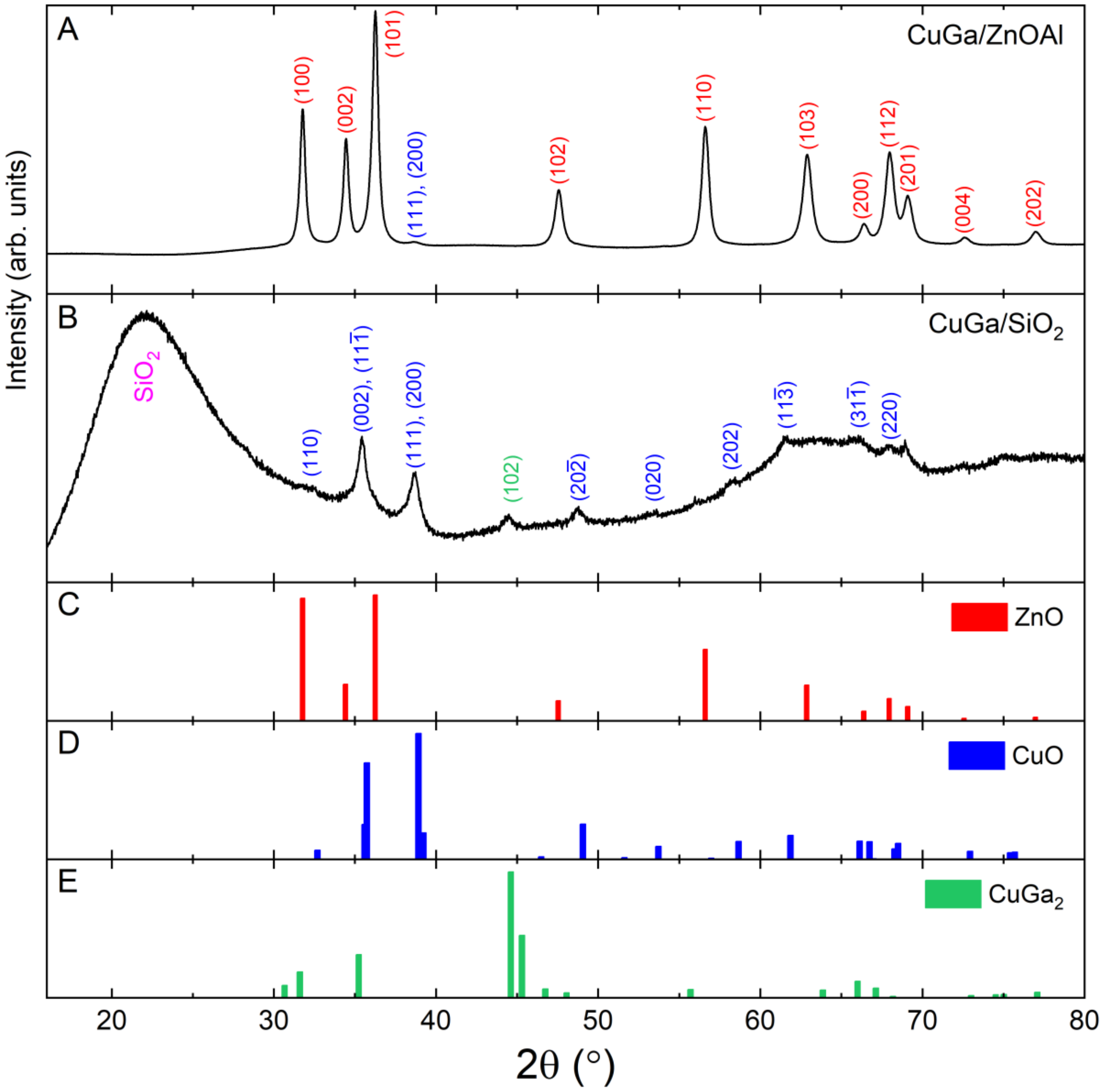

Fig. S1 XRD patterns for (A) CuGa/ZnOAl and (B) CuGa/SiO_2 nanocrystalline powder catalysts. Below the patterns of references (C) ZnO, (D) CuO and (E) $CuGa_2$ are shown.

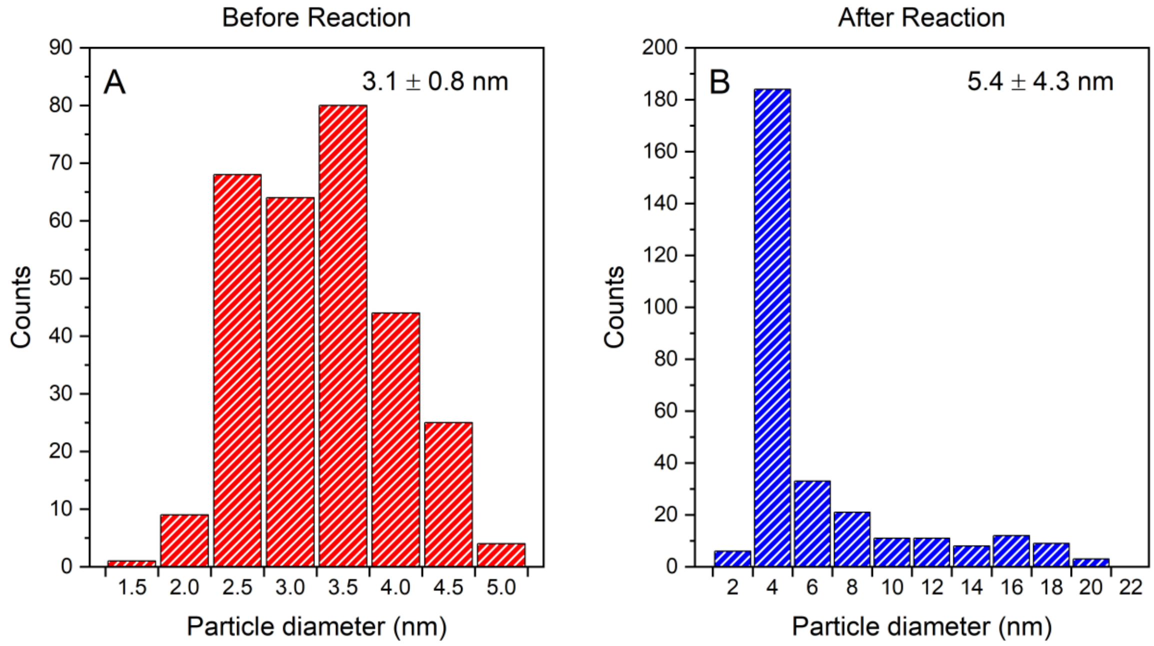

Fig. S2 Particle size histogram extracted from TEM images for the CuGa NPs supported on SiO_2 (A) before and (B) after the reaction.

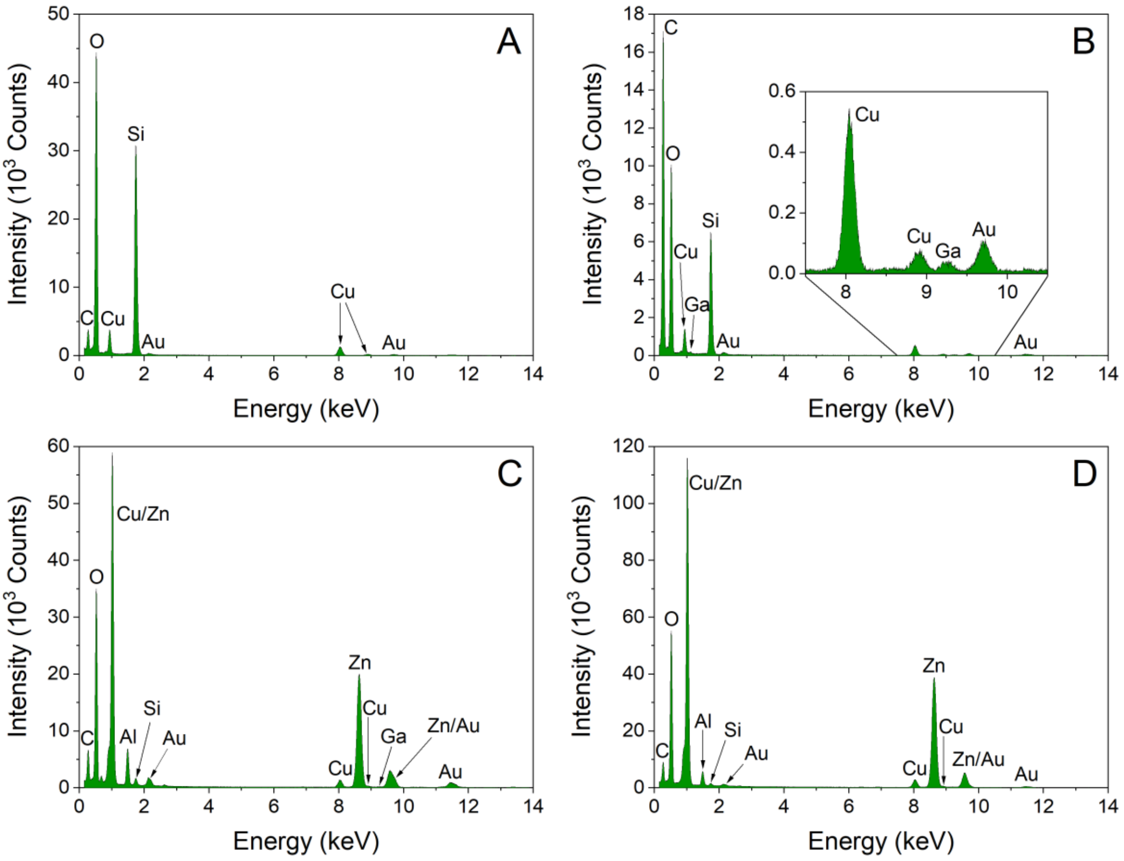

Fig. S3 EDX spectra of (A) nanocrystalline powder CuGa NP/SiO_2 catalyst in its initial state and (B) after the reaction. (C) CuGa NP/ZnOAl catalyst in its initial state and (D) after the reaction. Additional Au peaks originate from the grid used as support.

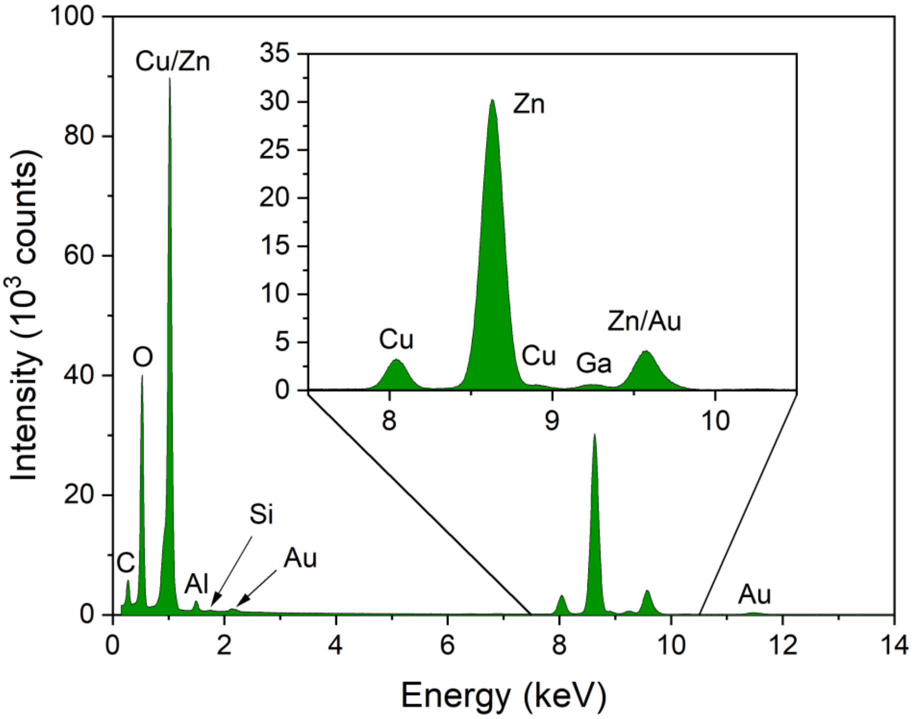

Fig. S4 EDX spectra of the CuGa/ZnOAl catalyst after the reaction (H_2 + CO_2, 250°C) (Figure 3). Additional Au peaks originate from the supporting grid.

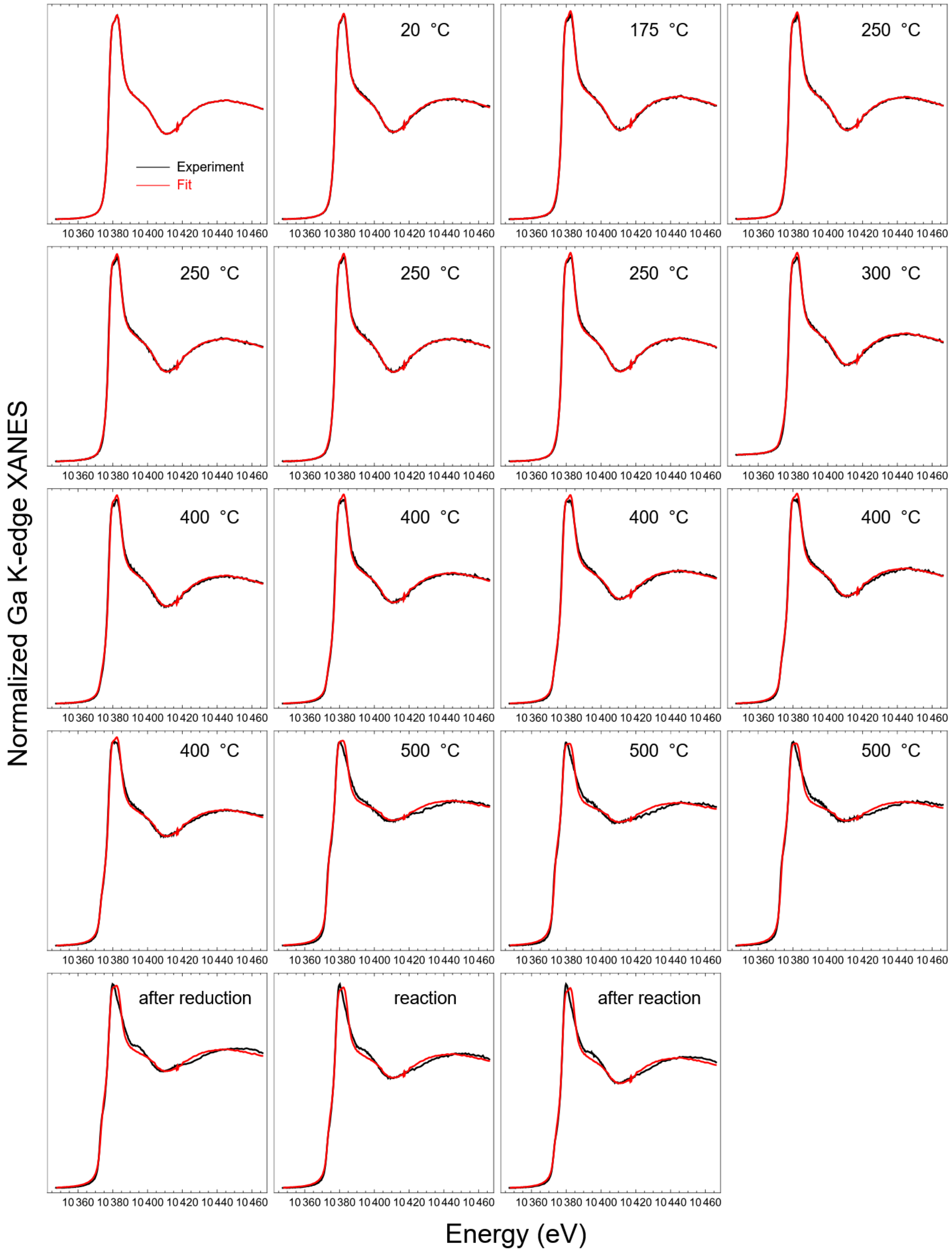

Fig. S5 Linear combination fits of the in-situ Ga K-edge XANES spectra, using the spectra for as-prepared catalyst in air (upper left figure), and the spectrum for metallic Ga as references.

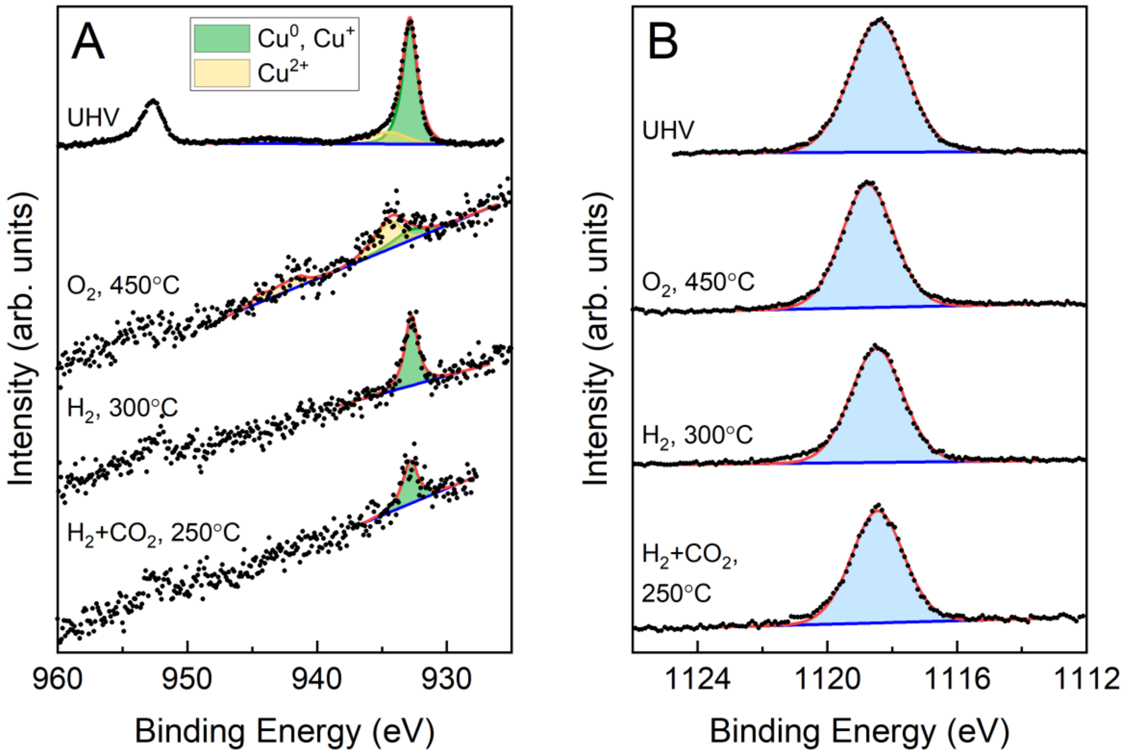

Fig. S6 Representative NAP-XPS spectra of the (A) Cu 2p and (B) Ga 2p regions for the $Cu_{70}Ga_{30}$ NPs supported on SiO_2/Si(111) measured with a photon energy of 1320 eV. The spectra shown here were acquired under ultra-high vacuum (UHV), oxidizing (450°C, O_2, 0.9 mbar), reducing (300°C, H_2, 1 mbar) and under reaction conditions (250°C, H_2 + CO_2 (3:1), 1 mbar).

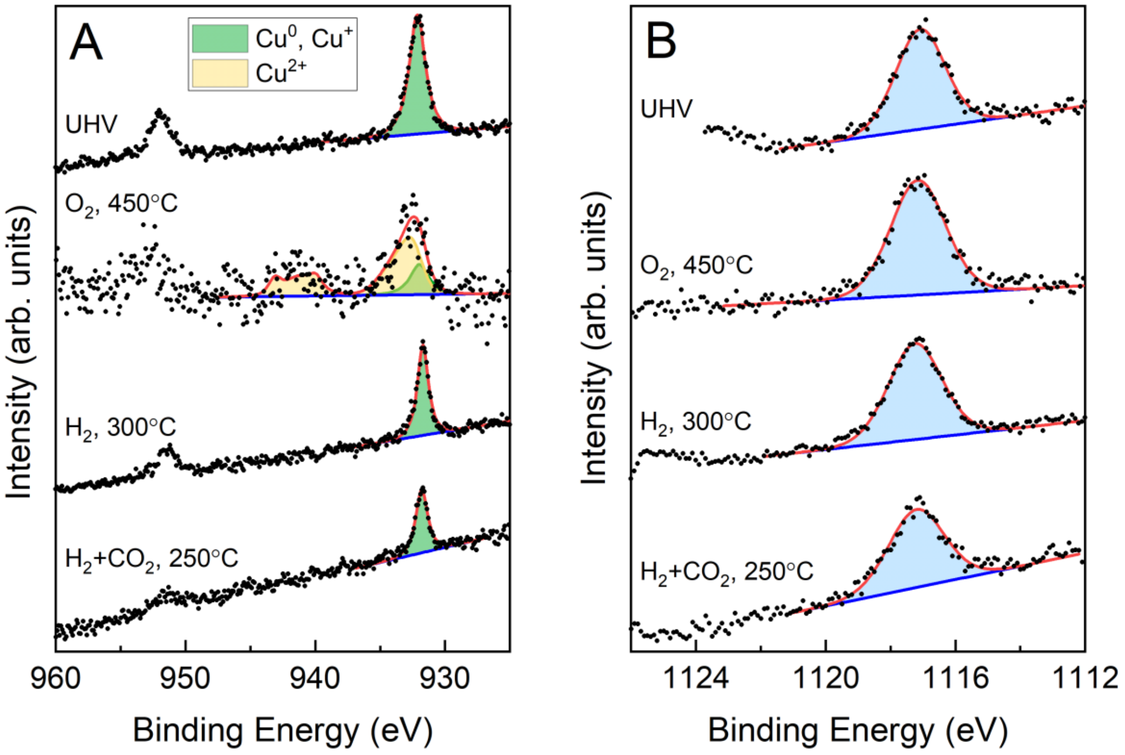

Fig. S7 Representative NAP-XPS spectra of the (A) Cu 2p and (B) Ga 2p regions for the $Cu_{70}Ga_{30}$ NPs supported on ZnO(0001) measured with a photon energy of 1320 eV. The spectra shown here were acquired under ultra-high vacuum (UHV), oxidizing (450°C, O_2, 0.9 mbar), reducing (300°C, H_2, 1 mbar) and reaction conditions (250°C, H_2 + CO_2 (3:1), 1 mbar).

Table S1 Atomic fraction of Cu and Ga in the as prepared (after calcination in O_2) catalysts obtained from ICP-MS.

Sample	Cu fraction (%)	Ga fraction (%)
CuGa/SiO_2	2.60 ± 0.01	0.301 ± 0.001
CuGa/ZnOAl	2.76 ± 0.02	0.323 ± 0.002

Table S2 EXAFS best fit parameters for Ga K-edge of a Ga_2O_3 reference and the CuGa/SiO_2 catalyst at different reaction steps. Uncertainties of the last digit are shown in parenthesis. Coordination numbers (N), average interatomic distances (R) and disorder factors (σ^2) for Ga-O and Ga-M (M = Cu or Ga) are reported, as well as the corrections to photoelectron reference energies (ΔE_0). EXAFS fitting is carried out in R-space, in the R-range between 1.15 and 3 Å, while the k-range between 2.5 and 10Å^{-1} was used for Fourier transform. S_0^2 factor was calculated to be equal to 1.4 from the analysis of reference spectra.

**Sample**	**N_{Ga-O}**	**R_{Ga-O} (Å)**	**σ^2_{Ga-O} (Å^2)**	**N_{Ga-M}**	**R_{Ga-M} (Å)**	**σ^2_{Ga-M} (Å^2)**	**ΔE_0 (eV)**	**Fit R-factor (%)**
**Ga_2O_3**	5	1.89(2)	0.012(3)	-	-	-	-3.6(1)	1.6
**CuGa (as prepared)**	3.8(2)	1.82(7)	0.007(1)	-	-	-	-1.8(9)	0.3
**CuGa (after reduction)**	2.0(4)	1.82(1)	0.005(3)	4.3(8)	2.61(1)	0.0092(6)	-8(2)	1.6
**CuGa (after reaction)**	2.6(4)	1.82(1)	0.005(2)	3.3(7)	262(1)	0.0092(6)	-6(2)	1.4

Table S3 XPS fit parameters for CuGa NPs/SiO_2/Si(111) and CuGa/ZnO(0001) under ultra-high vacuum (UHV), oxidizing (450°C, O_2, 0.9 mbar), reducing (300°C, H_2, 1 mbar) and reaction conditions (250°C, $H_2 + CO_2$ (3:1), 1 mbar). The nominal Cu:Ga ratio of the NPs before the reaction was about 60:40.

Sample	Condition	Cu/(Cu+Ga) (%)		Cu^{2+} fraction (%)	
		1320 eV	1540 eV	1320 eV	1540 eV
CuGa/SiO_2/Si(111)	UHV	57.1	57.1	26.0	26.0
	Oxidation	12.0 (2.0)	20.7 (2.0)	73.7	90.9
	Reduction	8.9 (0.6)	10.3 (2.1)	0	0
	Reaction	10.5 (1.7)	11.9 (1.0)	0	0
CuGa/ZnO(0001)	UHV	67.0	58.6	0	0
	Oxidation	37.3 (3.5)	48.2 (4.6)	80.9	97.4
	Reduction	33.8 (1.0)	35.2 (0.7)	0	0
	Reaction	42.4 (3.0)	38.5 (3.3)	0	0
